# Leucine rich repeat LGI family member 3: Integrative analyses support its prognostic association with pancreatic adenocarcinoma

**DOI:** 10.1097/MD.0000000000037183

**Published:** 2024-02-23

**Authors:** Hye-Young Yun

**Affiliations:** a Department of Biochemistry, Chung-Ang University, College of Medicine, Seoul, Republic of Korea.

**Keywords:** cancer, cytokine, LGI3, pancreatic adenocarcinoma, prognosis

## Abstract

Leucine rich repeat LGI family member 3 (LGI3) is a member of the LGI protein family. Previous studies of our group have reported that LGI3 is expressed in adipose tissue, skin and brain, and serves as a multifunctional cytokine. LGI3 may also be involved in cytokine networks in various cancers. This study aimed to analyze differentially expressed genes in pancreatic adenocarcinoma (PAC) tissues and PAC cohort data in order to evaluate the prognostic role of LGI3. The expression microarray and the PAC cohort data were analyzed by bioinformatic methods for differential expression, protein-protein interactions, functional enrichment and pathway analyses, gene co-expression network analysis, and prognostic association analysis. Results showed that LGI3 expression was significantly reduced in PAC tissues. Nineteen upregulated genes and 31 downregulated genes in PAC tissues were identified as LGI3-regulated genes. Protein-protein interaction network analysis demonstrated that 92% (46/50) of the LGI3-regulated genes that were altered in PACs belonged to a protein-protein interaction network cluster. Functional enrichment and gene co-expression network analyses demonstrated that these genes in the network cluster were associated with various processes including inflammatory and immune responses, metabolic processes, cell differentiation, and angiogenesis. PAC cohort analyses revealed that low expression levels of LGI3 were significantly associated with poor PAC prognosis. Analysis of favorable or unfavorable prognostic gene products in PAC showed that 93 LGI3-regulated genes were differentially associated with PAC prognosis. LGI3 expression was correlated with the tumor-infiltration levels of various immune cells. Taken together, these results suggested that LGI3 may be a potential prognostic marker of PAC.

## 1. Introduction

Leucine rich repeat LGI family member 3 (LGI3) is a secretory protein of the LGI family and is found in vertebrates.^[1]^ Expression of LGI3 in the brain was shown to be developmentally regulated at the transcription level by activating enhancer-binding protein 2 (AP-2) and neuronal restrictive silencer factor.^[1]^ LGI3 regulates neuronal exocytosis and differentiation^[2,3]^ and is also expressed in the epidermal layer of skin where it may act as a cytokine.^[4]^ Studies have shown that LGI3 was secreted in response to ultraviolet B (UVB) radiation and promoted the survival of keratinocytes.^[4]^ Additionally, it promotes the migration, differentiation, and inflammatory responses of keratinocytes^[5–9]^ and melanocyte pigmentation.^[10]^

LGI3 is expressed in adipose tissue and its expression is downregulated during adipocyte differentiation and upregulated in obese adipose tissue.^[11,12]^ Studies have also shown that LGI3 attenuated adipogenesis through a disintegrin and metalloproteinase domain-containing protein 23 (ADAM23), which is one of the LGI3 receptors (ADAM22 and ADAM23), and that LGI3 upregulated various pro-inflammatory genes including tumor necrosis factor-α (TNF-α) in macrophage cells,^[12]^ and downregulated adiponectin.^[11]^ LGI3 and TNF-α are mutually upregulated via NF-κB, suggesting that they play a cooperative role in regulating metabolic inflammation in obese individuals.^[13]^ LGI3 is also thought to be a pleiotropic cytokine and pro-inflammatory adipokine, which interplays with various cytokines, adipokines, chemokines, and signaling proteins.^[14,15]^

More recent studies have proposed that LGI3 may participate in the cytokine network in various cancers^[14,16,17]^ and that the expression levels of LGI3 could have potentially prognostic roles in brain, lung, and colorectal cancers.^[16–18]^ In this study, integrative analyses of gene expression, gene product networks, and patient cohorts were employed to present evidence for the potential prognostic role of LGI3 in pancreatic adenocarcinoma (PAC).

## 2. Materials and methods

### 2.1. Gene expression microarray data

The mRNA expression microarray datasets were retrieved from the Gene Expression Omnibus (GEO, http://www.ncbi.nlm.nih.gov/geo/) (Table 1). The GSE15471,^[19]^ GSE16515,^[20]^ GSE28735,^[21]^ GSE62452,^[22]^ and GSE77858 (unpublished) datasets were obtained from PAC tissues and paired adjacent normal tissues from patients. The GSE71729 dataset comprised 145 primary and 61 metastatic PAC tumors and 134 adjacent non-tumor tissues of pancreas^[23]^

**Table 1:** Datasets used in the study.

Dataset	Sample	Number	Platform
GSE15471	Normal	39	GPL570 [HG-U133_Plus_2] Affymetrix Human Genome U133 Plus 2.0 Array
	Tumor	39	
GSE16515	Normal	16	GPL570[HG-U133_Plus_2] Affymetrix Human Genome U133 Plus 2.0 Array
	Tumor	36	
GSE28735	Normal	45	GPL6244 [HuGene-1_0-st] Affymetrix Human Gene 1.0 ST Array
	Tumor	45	
GSE62452	Normal	61	GPL6244 [HuGene-1_0-st] Affymetrix Human Gene 1.0 ST Array
	Tumor	69	
GSE77858	Normal	38	GPL7264 Agilent-012097 Human 1A Microarray (V2) G4110B
	Tumor	46	
GSE71729	Normal	134	GPL20769 Agilent-014850 Whole Human Genome Microarray 4 × 44K G4112F
	Tumor	206	Primary tumor (n = 145), Metastasis tumor (n = 61)

Normal: adjacent non-tumor tissues of pancreas.

### 2.2. Data processing for identifying differentially expressed genes (DEGs)

The microarray datasets were analyzed by the affy package in R 4.2.1 (http://www.r-project.org).^[24]^ The datasets were subjected to background correction, quantile normalization, and probe summarization of expression values. The log2 intensities of probesets were calculated by the Robust Multichip Average (RMA) algorithm from the affy R package.^[24]^ Gene expression data were averaged to provide the final expression values for multiple probes for the same gene symbols, and the Affymetrix Microarray Suite 5 calls (MAS5CALLS) algorithm was used to exclude probesets to non-expressed mRNAs. Differential expression analysis was conducted by the limma package in R 4.2.1. Gene products with a *P* value of < .05 and a |log_2_ (fold change)| of ≥ 0.5 were considered statistically significant differentially expressed genes (DEGs).

### 2.3. Comparative analysis, protein-protein interaction network, functional enrichment, and gene co-expression network analyses

Venny 2.1 (http://bioinfogp.cnb.csic.es/tools/venny) was used to create a Venn diagram to comparatively analyze the categorized gene sets. The protein-protein interaction network was constructed by the data from the Search Tool for the Retrieval of Interacting Genes (STRING, version 11.5; http://string-db.org)^[25]^ and was visualized by Cytoscape 3.9.1 using the interaction degree-sorted circle layout.^[26]^ Functional enrichment analysis and Kyoto Encyclopedia of Genes and Genomes (KEGG) pathway analysis were conducted using the Database for Annotation, Visualization, and Integration Discovery (DAVID 2021; https://david.ncifcrf.gov),^[27]^ while the subsets of the entries with the lowest *P* values were also presented. Gene co-expression network (GCN) analysis was performed using the GCN of the human pancreas (uuid: 573fa3f6-5caf-11e7-8f50-0ac135e8bacf)^[28]^ obtained from the Network Data Exchange (NDex 2.5.3; http://www.ndexbio.org) and visualized by Cytoscape 3.9.1 using Prefuse force-directed layout. GCN analysis is used for elucidating the roles of gene sets because co-expressed genes are regulated by the common transcriptional programs and are members of the same protein complex or signaling pathway.^[29]^ Gene ontology (GO) categories of the GCN were mapped by BiNGO 3.0.5 and visualized by Cytoscape 3.9.1 using edge-weighted spring-embedded layout and hierarchical layout. PAC regulon was obtained from the Network Data Exchange (NDex 2.5.3; http://www.ndexbio.org; uuid: 4d0c897e-70c7-11e8-a4bf-0ac135e8bacf). The PAC regulon network was previously generated using the Algorithm for the Reconstruction of Accurate Cellular Networks (ARACNe) software package (https://califano.c2b2.columbia.edu/aracne)^[30]^ and The Cancer Genome Atlas (TCGA) data (https://portal.gdc.cancer.gov/projects/TCGA-PAAD) and was visualized by Cytoscape 3.9.1 using Prefuse force-directed layout. Transcriptional regulatory association between the groups of genes and transcription factors was assessed by transcription factor affinity prediction (TRAP) tools (http://trap.molgen.mpg.de).^[31]^ Correlations between tumor-infiltrating immune cells and expression of LGI3 was assessed by Tumor Immune Estimation Resource (TIMER 2.0; http://timer.cistrome.org).^[32]^

### 2.4. Meta-analysis of patient cohorts

The datasets of the gene expression microarray for PAC cohorts were retrieved from the Cancer Genome Atlas Program (TCGA; https://portal.gdc.cancer.gov/projects/TCGA-PAAD) and International Cancer Genome Consortium (ICGC; https://dcc.icgc.org). The clinicopathological data of the TCGA and ICGC cohorts are shown in Table S1, Supplemental Digital Content, http://links.lww.com/MD/L339. The datasets were previously processed using quality control tests, normalization, and batch effect adjustment while excluding low-quality samples. The correlation between gene expression values and PAC prognosis was assessed by the minimum *P* value method for survival analysis of patient groups, which calculates the cutoff point in continuous gene expression measurement. Patients ranked by gene expression values were dichotomized at the cutoff point to provide minimal *P* values and the difference in survival between high and low gene expression groups was calculated by the log-rank test. The statistically significant (*P* value < .05) datasets were used to generate Kaplan–Meier plots. The networks of scored correlations between genes and PAC were obtained from the Human Protein Atlas (HPA v21.0; https://www.ndexbio.org; DisGeNET, curated gene-disease associations; uuid: 904c6f47-38e4-11ec-b3be-0ac135e8bacf).^[33]^ The networks were constructed based on the immunohistochemistry profiles for cancer tissues and the log-rank *P* values for the Kaplan–Meier analysis of the correlation between mRNA expression level and patient survival. The data include Ensembl gene identifier, gene symbol, tumor name, the number of patients annotated for different staining levels (High, Medium, Low, Not detected), and the log-rank *P* values for patient survival and mRNA correlation. The positive and negative correlations between expression level and prognosis were annotated as favorable and unfavorable, respectively.

### 2.5. Statistical analysis

Significance was assessed using ANOVA with Bonferroni correction. The results were considered significant at *P* < .05. Statistical analyses were conducted using SPSS version 26 (IBM Corp.) and all statistical tests were two-sided. The hypergeometric test and Bonferroni correction were used to obtain *P* values in BiNGO analysis.

## 3. Results

### 3.1. Differential expression of LGI3 in PAC

**Figure 1.**
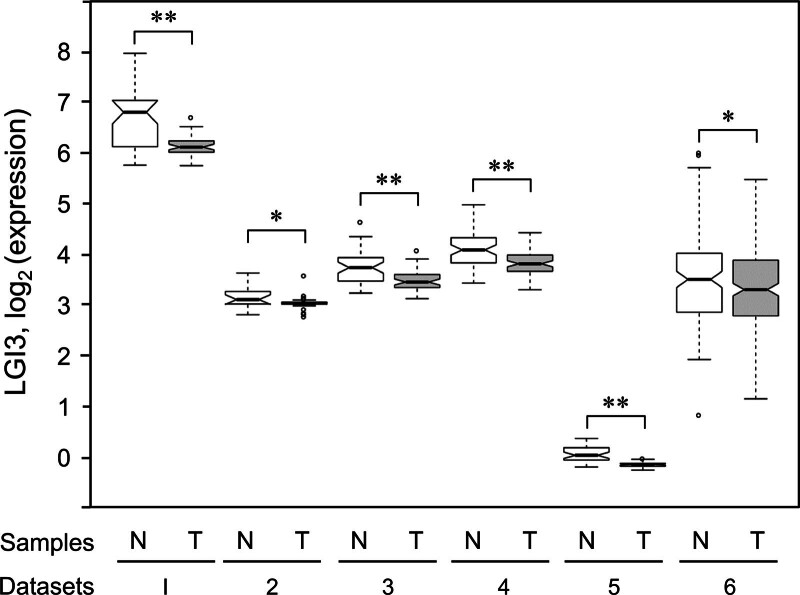
Differential expression of LGI3 in PAC tissues. Open bar, control (non-tumor) tissues; shaded bar, PAC tumor tissues; N, non-tumor; T, tumor. Datasets 1, GSE15471; 2, GSE16515; 3, GSE28735; 4, GSE62452; 5, GSE77858; 6, GSE71729 (Table 1). **P < .001; *P < .05.

Previous studies reported that expression levels of LGI3 were decreased in cases of glioma and non-small cell lung cancer (NSCLC).^[16–18]^ In this study, analysis of the DEGs in the pancreas tissues taken from PAC patients showed that LGI3 expression in tumor tissues was significantly lower than in healthy tissue (FC, fold change; FC = 0.70, *P* = 1.23 × 10^−7^ in GSE15471; FC = 0.91, *P* = 3.6 × 10^−2^ in GSE16515; FC = 0.85, *P* = 4.82 × 10^−5^ in GSE28735; FC = 0.83, *P* = 1.20 × 10^−6^ in GSE62452; FC = 0.88, *P* = 1.37 × 10^−13^ in GSE77858; FC = 0.89, *P* = 4.48 × 10^−2^ in GSE71729) (Fig. 1).

### 3.2. Identification of LGI3-regulated and PAC-altered genes and their protein-protein interaction networks

**Figure 2.**
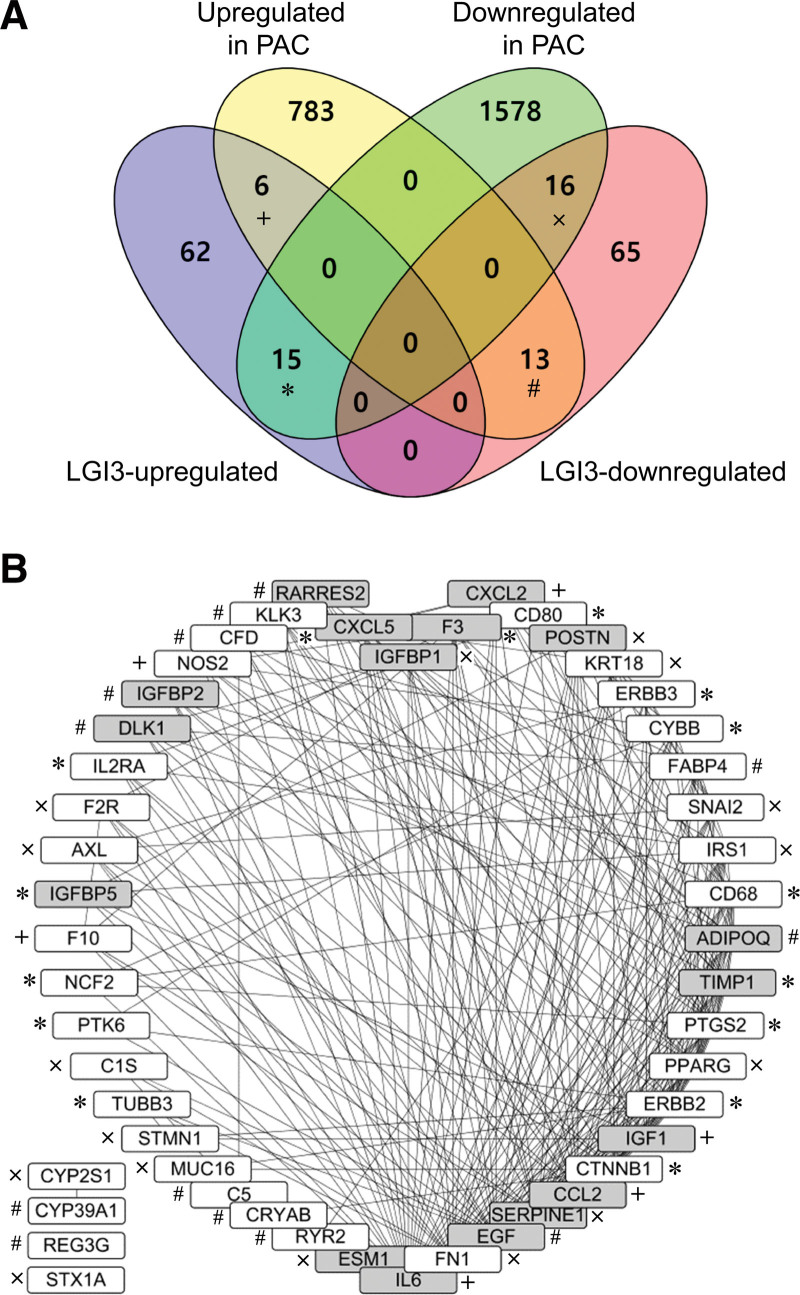
Comparative analysis of the up- or downregulated genes in PAC and LGI3-regulated genes. (A) Venn diagram showing the sets of the regulated gene categories. (B) Protein-protein interaction network of PAC-altered and LGI3-regulated products. The network was depicted by nodes (gene products) and lines (pairwise protein interactions) sorted by interaction degrees. The marks (*, +, #, x) indicate the gene products in the common sets of the regulated gene categories indicated in Figure 2A. Gray nodes indicate cytokines, adipokines, and chemokines.

Analyses of DEGs in the 6 expression microarray PAC datasets showed that 802 gene products were increased, and 1609 gene products were decreased in the PAC tissues (|log_2_ FC|≥0.5 and *P* < .05 in ≥ 2 datasets; Table S2, Supplemental Digital Content, http://links.lww.com/MD/L340). Our previous studies identified 177 gene products that were regulated by LGI3 (Table S3, Supplemental Digital Content, http://links.lww.com/MD/L341).^[14,15]^ Venn diagram analysis of PAC-altered genes and LGI3-regulated genes showed that 6 PAC-upregulated genes and 15 PAC-downregulated genes were identified as LGI3-upregulated genes, while 13 PAC-upregulated genes and 16 PAC-downregulated genes were identified as LGI3-downregulated genes (Fig. 2A; Table S4, Supplemental Digital Content, http://links.lww.com/MD/L342). The expression of 25% (21/83) of LGI3-upregulated genes and 31% (29/94) of LGI3-downregulated genes were dysregulated in PAC tissues. Protein-protein interaction network analysis of 50 PAC-altered and LGI3-regulated genes demonstrated that 92% (46/50) of the gene products formed an interaction network cluster (Fig. 2B). In addition, 17 of the gene products from among the 46 LGI3-regulated and PAC-altered gene products in a protein-protein interaction network cluster (Fig. 2B) were identified as cytokines. The 17 cytokines were adiponectin (ADIPOQ), C-C motif chemokine ligand 2 (CCL2), C-X-C motif chemokine ligand 2 (CXCL2), C-X-C motif chemokine ligand 5 (CXCL5), delta-like non-canonical Notch ligand 1 (DLK1), epidermal growth factor (EGF), endothelial cell-specific molecule 1 (ESM1), coagulation factor III, tissue factor (F3), insulin-like growth factor 1 (IGF1), insulin-like growth factor binding protein 1 (IGFBP1), insulin-like growth factor binding protein 2 (IGFBP2), insulin-like growth factor binding protein 5 (IGFBP5), interleukin 6 (IL6), periostin (POSTN), retinoic acid receptor responder 2 (RARRES2), serpin family E member 1 (SERPINE1), and TIMP metallopeptidase inhibitor 1 (TIMP1) (Fig. 2B, gray nodes). The proteins with the highest degrees of interaction (≥20) were interleukin 6 (IL6), fibronectin 1 (FN1), EGF, serpin family E member 1 (SERPINE1), C-C motif chemokine ligand 2 (CCL2, MCP-1), catenin beta 1 (CTNNB1), insulin-like growth factor 1 (IGF1), erb-b2 receptor tyrosine kinase 2 (ERBB2), peroxisome proliferator-activated receptor γ (PPARG), and prostaglandin-endoperoxide synthase 2 (PTGS2, COX2).

### 3.3. Functional enrichment analyses of LGI3-regulated and PAC-altered genes

The functional signature of LGI3-regulated gene products that are altered in PAC tissue was identified by using functional enrichment analysis to investigate the GO of the gene groups (Table 2). The gene groups were significantly associated with inflammatory and immune responses including lipopolysaccharide response, cytokines, insulin-like growth factor, and chemokine activities. The KEGG pathway analysis of LGI3-regulated and PAC-altered genes also demonstrated that the gene groups were associated with the pathways of hypoxia-inducible factor-1 (HIF-1), TNF, cytokines, infectious diseases, and cancer-related pathways (Table 3). A majority of the associated KEGG pathways were also found to be related to inflammatory and immune systems.

**Table 2:** Functional enrichment analysis of leucine rich repeat LGI family member 3-regulated genes that are altered in pancreatic adenocarcinoma.

Category	Term	Count	*P* value
GOTERM_CC_DIRECT	GO:0005615~extracellular space	26	1.42 × 10^−13^
GOTERM_CC_DIRECT	GO:0005576~extracellular region	23	6.41 × 10^−10^
GOTERM_BP_DIRECT	GO:0006954~inflammatory response	12	5.15 × 10^−9^
GOTERM_BP_DIRECT	GO:0071222~cellular response to lipopolysaccharide	9	2.79 × 10^−8^
GOTERM_BP_DIRECT	GO:0008284~positive regulation of cell proliferation	12	9.99 × 10^−8^
GOTERM_CC_DIRECT	GO:0031093~platelet alpha granule lumen	6	5.50 × 10^−7^
GOTERM_BP_DIRECT	GO:0032757~positive regulation of interleukin-8 production	6	6.37 × 10^−7^
GOTERM_MF_DIRECT	GO:0005102~receptor binding	10	7.24 × 10^−7^
GOTERM_BP_DIRECT	GO:0007165~signal transduction	14	1.15 × 10^−5^
GOTERM_MF_DIRECT	GO:0005520~insulin-like growth factor binding	4	2.84 × 10^−5^
GOTERM_MF_DIRECT	GO:0005515~protein binding	46	3.65 × 10^−5^
GOTERM_BP_DIRECT	GO:0010628~positive regulation of gene expression	9	4.14 × 10^−5^
GOTERM_BP_DIRECT	GO:0043410~positive regulation of MAPK cascade	6	5.28 × 10^−5^
GOTERM_BP_DIRECT	GO:0014068~positive regulation of phosphatidylinositol 3-kinase signaling	5	5.87 × 10^−5^
GOTERM_CC_DIRECT	GO:0005788~endoplasmic reticulum lumen	7	8.65 × 10^−5^
GOTERM_BP_DIRECT	GO:0007568~aging	6	1.31 × 10^−4^
GOTERM_CC_DIRECT	GO:0070062~extracellular exosome	16	1.39 × 10^−4^
GOTERM_BP_DIRECT	GO:0043567~regulation of insulin-like growth factor receptor signaling pathway	3	1.76 × 10^−4^
GOTERM_MF_DIRECT	GO:0031995~insulin-like growth factor II binding	3	1.84 × 10^−4^
GOTERM_BP_DIRECT	GO:0032355~response to estradiol	5	1.88 × 10^−4^
GOTERM_BP_DIRECT	GO:0042593~glucose homeostasis	5	2.01 × 10^−4^
GOTERM_BP_DIRECT	GO:0014065~phosphatidylinositol 3-kinase signaling	4	2.04 × 10^−4^
GOTERM_BP_DIRECT	GO:0009617~response to bacterium	5	2.53 × 10^−4^
GOTERM_BP_DIRECT	GO:0006935~chemotaxis	5	2.69 × 10^−4^
GOTERM_MF_DIRECT	GO:0008009~chemokine activity	4	2.99 × 10^−4^

BP = biological process, CC = cellular component, GO = gene ontology, MF = molecular function.

**Table 3:** Kyoto Encyclopedia of Genes and Genomes pathway analysis of leucine rich repeat LGI family member 3-regulated genes that are altered in pancreatic adenocarcinoma.

Term	Count	*P* value
hsa04066:HIF-1 signaling pathway	8	6.78 × 10^−7^
hsa04610:Complement and coagulation cascades	7	2.66 × 10^−6^
hsa05200:Pathways in cancer	12	2.52 × 10^−5^
hsa01521:EGFR tyrosine kinase inhibitor resistance	6	3.34 × 10^−5^
hsa04933:AGx10-RAGE signaling pathway in diabetic complications	6	1.04 × 10^−4^
hsa04151:PI3K-Akt signaling pathway	9	2.19 × 10^−4^
hsa05133:Pertussis	5	4.47 × 10^−4^
hsa05323:Rheumatoid arthritis	5	9.60 × 10^−4^
hsa04657:IL-17 signaling pathway	5	1.00 × 10^−3^
hsa05215:Prostate cancer	5	1.12 × 10^−3^
hsa04061:Viral protein interaction with cytokine and cytokine receptor	5	1.26 × 10^−3^
hsa04668:TNF signaling pathway	5	1.91 × 10^−3^
hsa05417:Lipid and atherosclerosis	6	3.37 × 10^−3^
hsa04936:Alcoholic liver disease	5	4.52 × 10^−3^
hsa05171:Coronavirus disease – COVID-19	6	4.66 × 10^−3^
hsa04020:Calcium signaling pathway	6	5.38 × 10^−3^
hsa05140:Leishmaniasis	4	5.79 × 10^−3^
hsa05022:Pathways of neurodegeneration - multiple diseases	8	6.65 × 10^−3^
hsa04211:Longevity regulating pathway	4	8.65 × 10^−3^
hsa05010:Alzheimer disease	7	9.14 × 10^−3^

### 3.4. Gene co-expression network analysis of LGI3-regulated and PAC-altered genes

**Figure 3.**
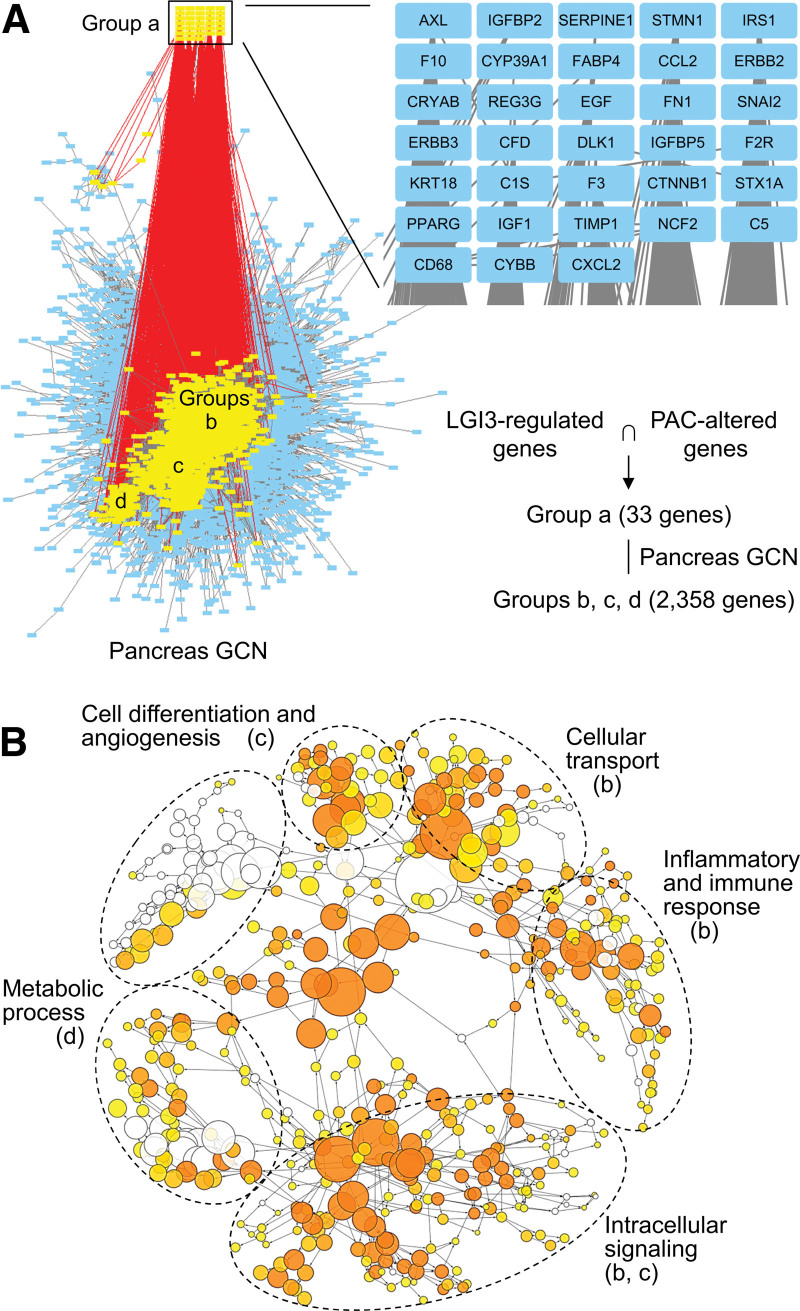
Gene co-expression network analysis in the pancreas of PAC-altered and LGI3-regulated gene products. (A) PAC-altered and LGI3-regulated gene products found in the pancreas GCN (Group a) and the subnetwork of the gene group (Groups b, c, and d) co-expressed with the group a genes. GCN, gene co-expression network. (B) Gene ontology map of the subnetwork consisting of the genes in groups b, c, and d. Letters in parentheses indicate the groups of the subnetwork in Figure 3A.

The gene set was queried against the GCN of the pancreas in order to investigate the roles in the pancreas of LGI3-regulated genes that are altered in PAC. In total, 33 gene products in the gene set (Fig. 2B) were found in the pancreas GCN (Fig. 3A, group a) and were associated with 2358 gene products in the network (Fig. 3A, groups b, c, and d). The subnetwork of co-expression with 2358 gene products consisted of a domain with 3 adjacent subnetwork clusters in the pancreas GCN (Fig. 3A, groups b, c, and d). The GO category map of the subnetworks (Groups b, c, and d, Figure 3A and B) revealed that the gene products contained within the networks are associated with inflammatory and immune responses (Group b), cell differentiation and angiogenesis (Group c), metabolic processes (Group d), cellular transport (Group b) and intracellular signaling (Groups b and c). The TRAP of the genes in the pancreas GCN subnetwork (Groups b, c, and d) associated with LGI3-regulated genes suggested that these genes may be co-expressed under the common transcriptional regulatory programs by various transcription factors (Chch, Tfii-i, Dp-1, E2f-1, Sp1, Maz, Ap-2, Movo-b, Fbi-1, Zf5, etc.) (Table S5, Supplemental Digital Content, http://links.lww.com/MD/L343).

### 3.5. Analysis of LGI3-regulated genes that are altered during PAC metastasis

**Figure 4.**
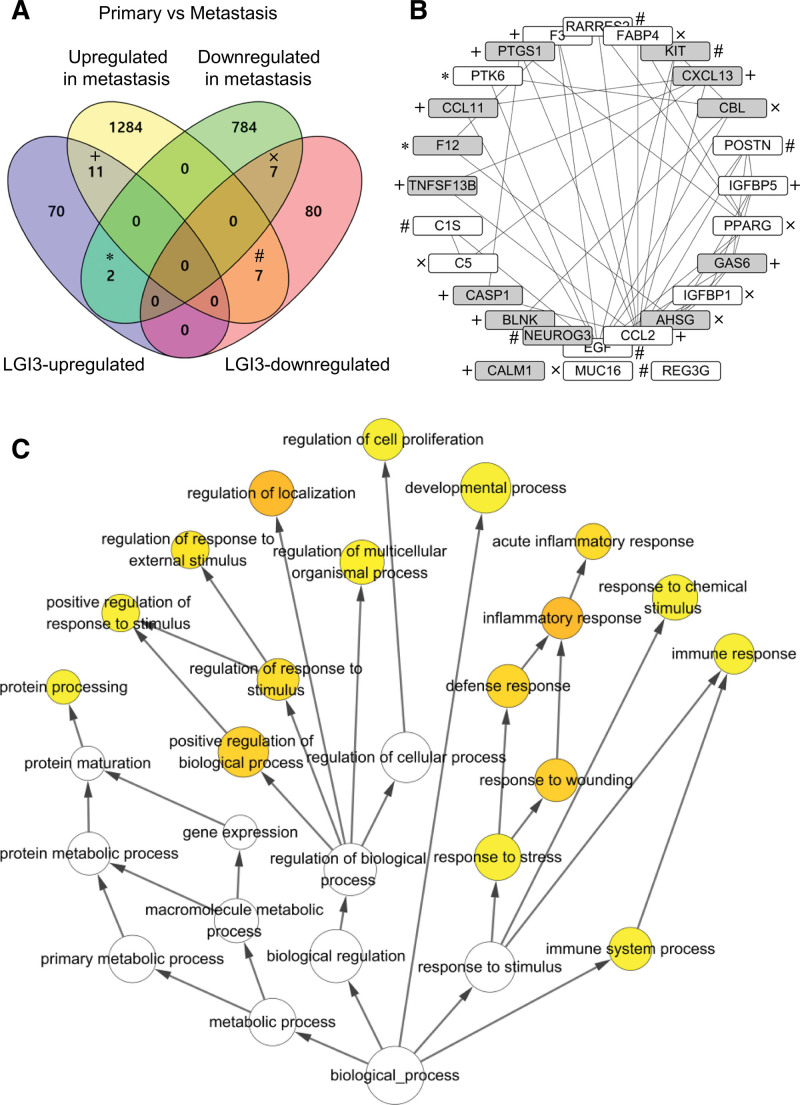
Comparative analysis of the up- or downregulated genes in PAC metastasis comparing with the primary PAC and LGI3-regulated genes. (A) Venn diagram showing the sets of regulated gene categories. (B) Protein-protein interaction network of metastasis-altered and LGI3-regulated gene products. The network is depicted by nodes (gene products) and lines (pairwise protein interactions) sorted by interaction degrees. The marks (+, x, *, #) indicate the gene products in the common sets of the regulated gene categories indicated in Figure 4A. Gray nodes show the metastasis-specific PAC-altered and LGI3-regulated genes that were not significantly changed in primary tumor compared with normal tissues. (C) Gene ontology map of the interaction network in Figure 4B. The nodes represent GO terms and arrow lines indicate parental relationships in the network hierarchy. The darker nodes represent lower *P* values in BiNGO analysis and indicate statistically more overrepresented GO terms (*P* < .01).

To explore the roles of LGI3-regulated gene products in the metastasis of PAC, the dataset (GSE71729) containing mRNA expression data from the primary (n = 145) and metastatic tumor (n = 61) was analyzed. Comparative analysis using a Venn diagram showed that 11 metastasis-upregulated genes and 2 metastasis-downregulated genes were identified as LGI3-upregulated genes, while 7 metastasis-upregulated genes and 7 metastasis-downregulated genes were identified as LGI3-downregulated genes (Fig. 4A, Table S6, Supplemental Digital Content, http://links.lww.com/MD/L344). Thirteen gene products were found to be metastasis-specific, PAC-altered, LGI3-regulated genes (AHSG, BLNK, CALM1, CASP1, CBL, CCL11, CXCL13, F12, GAS6, KIT, NEUROG3, PTGS1, TNFSF13B; Figure 4B, gray nodes) in that these genes were not significantly altered in the primary tumor compared with healthy tissue. Protein-protein interaction network analysis of 27 metastasis-altered and LGI3-regulated genes revealed that 89% (24/27) of the gene products formed an interaction network cluster (Fig. 4B). The GO category map of the interaction network indicated that the gene products in the network are associated with immune and inflammatory response, cell proliferation, development, localization, and protein processing (Fig. 4C).

### 3.6. Analysis of the PAC regulon

**Figure 5.**
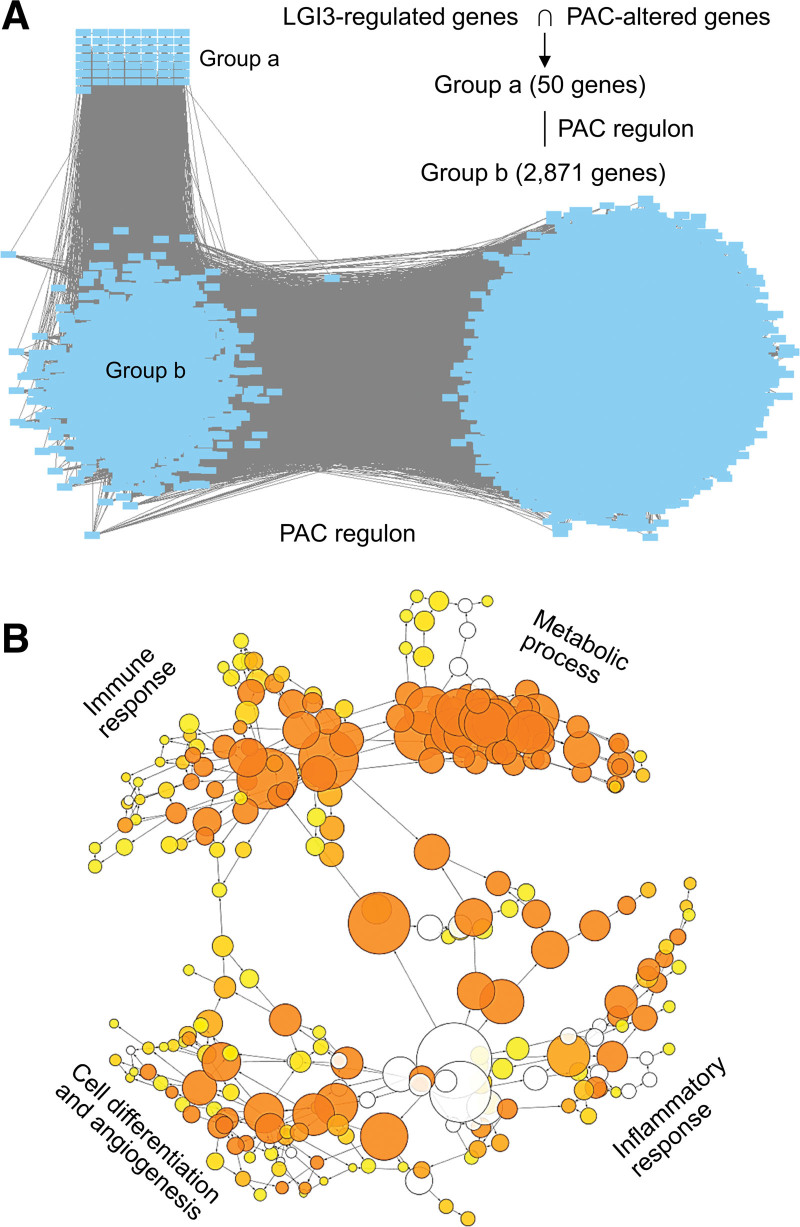
PAC regulon network analysis PAC-altered and LGI3-regulated gene products. (A) PAC-altered and LGI3-regulated gene products found in PAC regulon (Group a) and the subnetwork of the gene group b co-regulated with group a genes. (B) Gene ontology map of the subnetwork consisting of the genes in group b.

The association of LGI3-regulated gene products in the PAC regulatory network was investigated using the PAC regulon constructed by ARACNe.^[30]^ The PAC regulon used microarray expression profiles and an information-theoretic approach to eliminate the majority of indirect interactions.^[34]^ Altogether, 50 LGI3-regulated genes that are changed in PAC were identified in the PAC regulon (Fig. 5A, Group a). This gene group was found to be primarily associated with 2871 gene products in the PAC regulon (Fig. 5A, group b). The TRAP of this particular gene group (Fig. 5A, group b) indicated that these genes may be regulated by a group of transcription factors (Sp1, Sp2, Ap-2α/γ, Maz, Dp-1, E2f-1, Spz1, Fbi-1, Lrf, Movo-b, Egr-1/2/3/4, Zf5, Egr-1, Deaf-1, Tfii-I, Creb, Mazr, Chch, Atf-1/2, and C-jun; *P* < .001). The GO category map of the PAC regulon subnetwork with groups a and b (Fig. 5B) showed that the gene products in the networks are significantly associated with inflammatory and immune responses, cell differentiation, angiogenesis and metabolic processes.

### 3.7. Association of LGI3 expression with the prognosis of PAC

**Figure 6.**
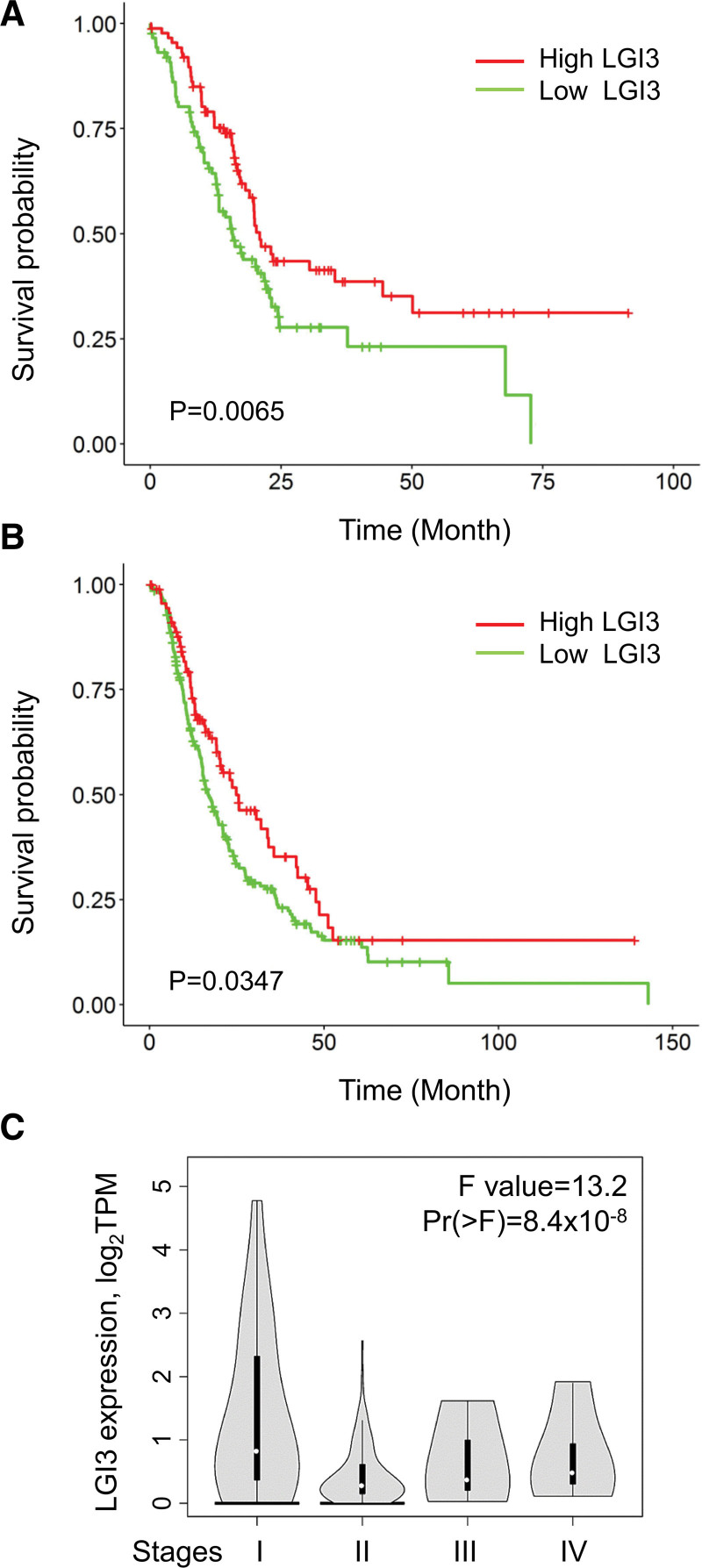
Associations of LGI3 expression with the prognosis of PAC patient cohorts. (A and B) Kaplan–Meier curves of the datasets of PAC from the cohorts of TCGA (A) and ICGC (B). (C) Expression of LGI3 mRNA in PAC stages.

The downregulation of LGI3 expression in PAC tissues (Fig. 1) implied the involvement of LGI3 with the morbidity and mortality of PAC. In order to assess the prognostic significance of LGI3 expression in PAC, the TCGA and ICGC data of PAC patient cohorts were analyzed, with the results showing that low expression of LGI3 was significantly associated with poor prognosis of PAC (Fig. 6A and B). Both overall survival and relapse-free survival were correlated with LGI3 expression. Comparing LGI3 expression in PAC stages revealed that LGI3 decreased most prominently between stages I and II (Fig. 6C).

### 3.8. Analysis of LGI3-regulated gene products that are prognostic for PAC

**Figure 7.**
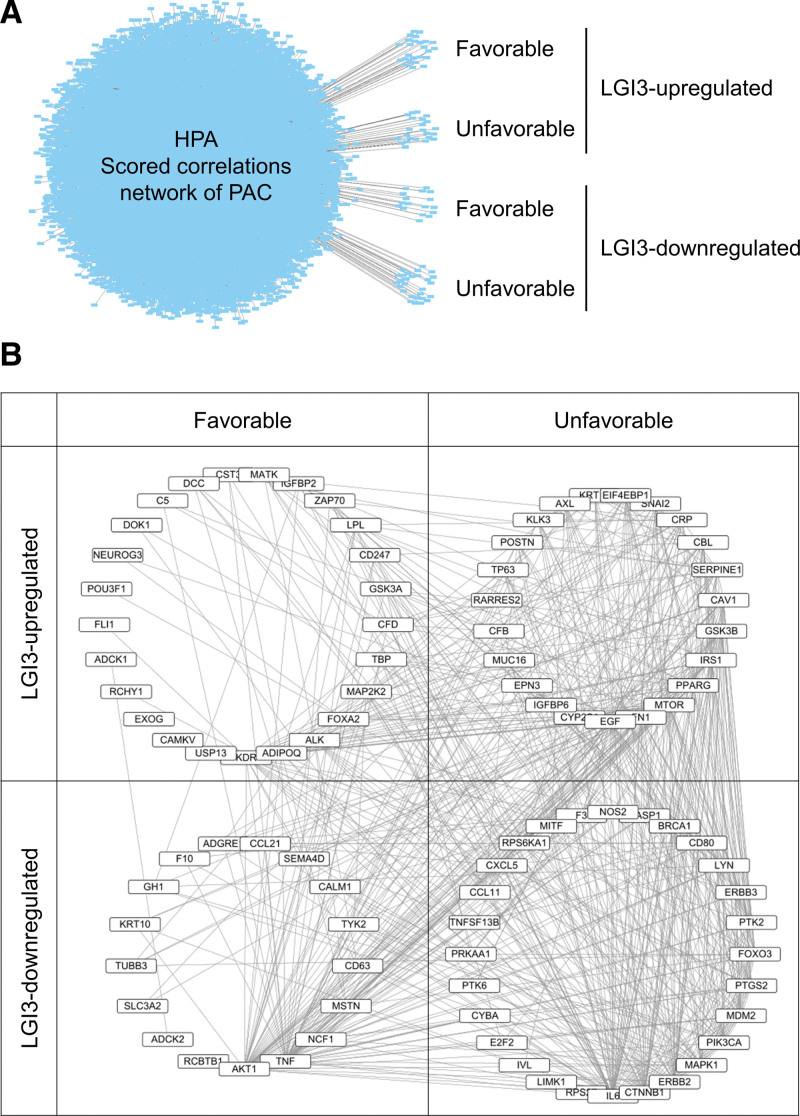
Association of LGI3-regulated gene products in the scored correlations network of PAC prognosis. (A) The scored correlations network of prognostically favorable or unfavorable genes that are regulated by LGI3. (B) Protein-protein interaction network of LGI3-regulated and PAC-prognostic genes. The network is depicted by the categorized subnetworks with nodes (gene products) and lines (pairwise protein interactions) sorted by interaction degrees.

The prognostic association of LGI3-regulated gene products and their interaction network with PAC was investigated using the gene-disease association networks of scored correlations between genes and PAC.^[33]^ The results showed that 25 prognostically favorable genes and 23 unfavorable genes were identified as LGI3-upregulated genes, while 17 favorable genes and 28 unfavorable genes were identified as LGI3-downregulated genes (Fig. 7A, Table S7, Supplemental Digital Content, http://links.lww.com/MD/L346). All the prognostic gene products regulated by LGI3 appeared to form a protein-protein interaction network cluster with 4 categorial subnetworks (Fig. 7B). Functional enrichment analysis of the subnetworks suggested that each subnetwork of LGI3-regulated and PAC-prognostic genes is involved in both overlapping and differential biological functions (Table S8, Supplemental Digital Content, http://links.lww.com/MD/L348). Notably, the programmed cell death-1 (PD-1) checkpoint pathway, EGFR tyrosine kinase inhibitor resistance, and various inflammatory functions were found to be associated with LGI3-regulated and PAC-prognostic genes (Table S8, Supplemental Digital Content, http://links.lww.com/MD/L348).

### 3.9. The correlation between LGI3 expression and tumor-infiltrating immune cells

**Figure 8.**
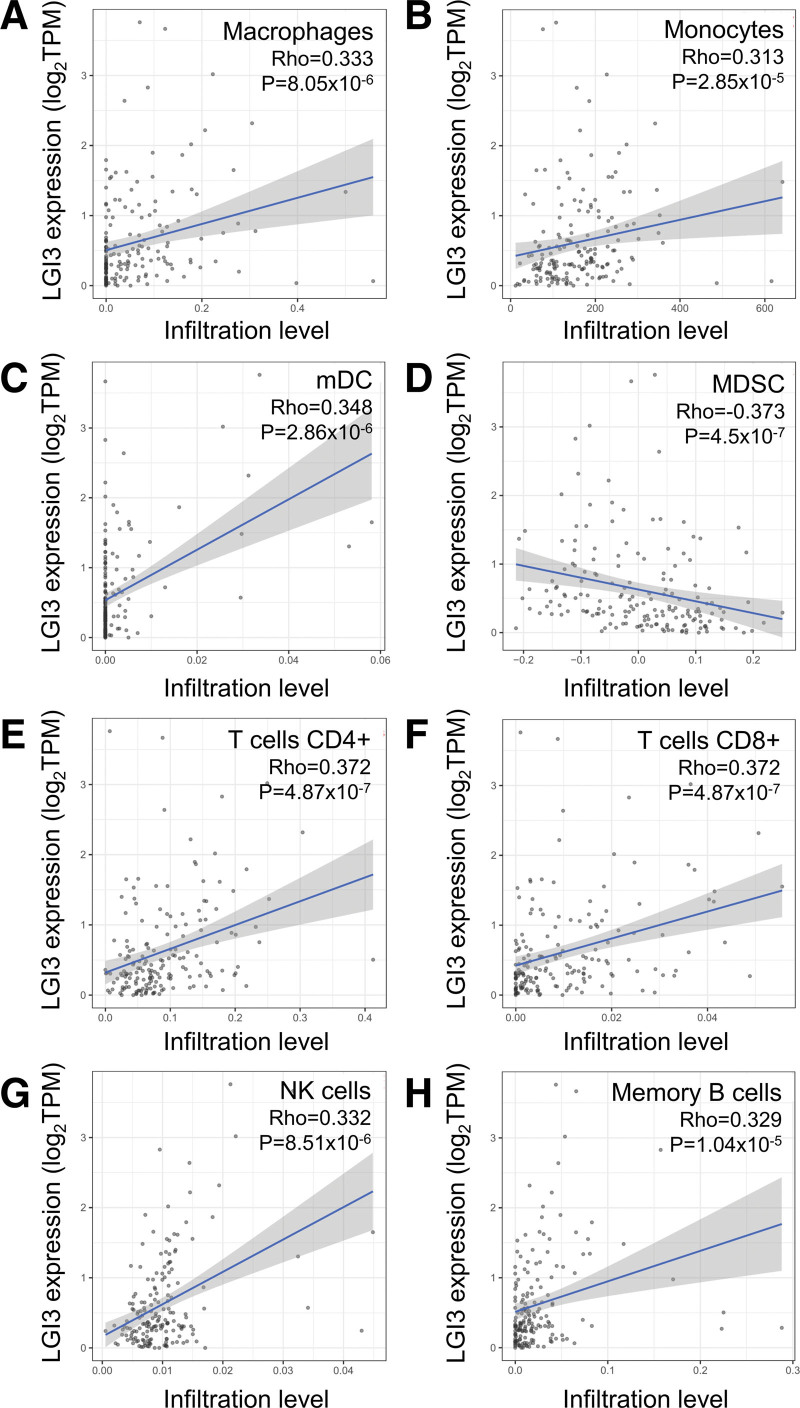
The correlation of LGI3 expression and immune infiltration in PAC. mDC = myeloid dendritic cells, MDSC = myeloid-derived suppressor cells, NK = natural killer.

Analysis of tumor-immune infiltrations in PAC by TIMER showed that infiltration levels of macrophages, monocytes, myeloid dendritic cells (mDC), CD4 + T cells, CD8 + T cells, natural killer cells and memory B cells were positively correlated with LGI3 expression significantly (Fig. 8A, B, C, E, F, G, and H). The infiltration level of myeloid-derived suppressor cells was negatively associated LGI3 expression (Fig. 8D).

## 4. Discussion

The LGI family genes (LGI1, 2, 3, 4) are expressed in various tumor cells and their expression is not correlated with the normal cell origins.^[35]^ The first gene member, LGI1, was proposed to be a tumor suppressor in brain tumors.^[35,36]^ Expression levels and genetic variations of LGI3 were postulated to have prognostic value in brain, colorectal, and lung cancer.^[16–18]^ The association of low expression levels of LGI3 with a negative prognosis of glioma and NSCLC^[17,18]^ suggested that dysregulated expression of LGI3 may affect the cytokine network in cancer progression.^[14,16]^

The present study explored the potential prognostic value and functional network of LGI3 in PAC using integrative analysis of transcriptomics data and LGI3-regulated cytokine networks.^[14,16]^ The significant reduction in LGI3 expression in PAC tissue (Fig. 1) suggested that the LGI3 signaling pathway may be perturbed in PAC carcinogenesis and progression. The components of intracellular signaling of LGI3 identified in the previous studies were Akt and FAK in neurite outgrowth,^[3]^ p53 and MDM2 in keratinocyte protection under UVB radiation,^[4]^ GSK3β and β-catenin in keratinocyte migration,^[5]^ microphthalmia-associated transcription factor (MITF) in melanogenesis,^[10]^ and PPARγ, C/EBPα, and NF-κB in adipogenesis and inflammation.^[12,13]^ LGI3 was found to regulate various signaling proteins in preadipocytes (Akt, AMPK, Erk, and PTEN were upregulated; 4E-BP1, Bad, and GSK3α were downregulated).^[14]^ However, the mediators of LGI3-induced signaling pathways that are active and dysregulated in PAC cells still need to be determined.

Previous studies reported that various gene products were regulated by LGI3.^[1–5,10–15]^ Most LGI3-regulated gene products (169 out of 177) were also found to form a protein-protein interaction network cluster.^[15]^ Fifteen genes (CD68, CD80, CTNNB1, CXCL5, CYBB, ERBB2, ERBB3, F3, IGFBP5, IL2RA, NCF2, PTGS2, PTK6, TIMP1, and TUBB3) that were reportedly upregulated by LGI3 may be decreased in PAC because LGI3 is already downregulated (Fig. 2A, *). Conversely, 13 genes (ADIPOQ, C5, CFD, CRYAB, CYP39A1, DLK1, EGF, FABP4, IGFBP2, KLK3, RARRES2, REG3G, and RYR2) that were shown to be downregulated by LGI3 may be increased in PAC, again because LGI3 is downregulated in PAC (Fig. 2A, #). It can be postulated, therefore, that the perturbated expression in PAC of these 2 gene groups (Fig. 2A, * and #) was predominantly affected by LGI3 downregulation in PAC. LGI3 may functionally interact with these gene products via a protein-protein interaction network (Fig. 2B) and through the mechanisms suggested by the functional enrichment and KEGG pathway analyses (Tables 2 and 3). Notably, the LGI3-regulated protein-protein interaction network included various cytokines, adipokines, or chemokines, suggesting that LGI3 plays a critical role in cytokine networks.^[14]^ In total, 17 cytokines, adipokines, or chemokines out of the 46 LGI3-regulated and PAC-altered gene products could significantly influence the protein-protein interaction network cluster (Fig. 2B), implying that an LGI3-regulated cytokine network may dysregulate intercellular communication in the PAC microenvironment.

Both functional enrichment analyses and GCN analysis of the LGI3-regulated and PAC-altered genes revealed a significant association with inflammatory and immune responses, as well as cytokine and chemokine activity (Tables 2 and 3; Fig. 3). LGI3-regulated cytokine networks may play pivotal roles in the inflammatory and immune responses in the PAC microenvironment.

The expression, genetic variations, and functions of all the LGI3-regulated and PAC-altered cytokines, adipokines, and chemokines (Fig. 2B, gray nodes) are all associated with PAC. The dysregulated expression levels of ADIPOQ,^[37]^ CCL2,^[38]^ DLK1,^[39]^ ESM1,^[40]^ F3,^[41]^ IGF1,^[42]^ IGFBP1,^[43]^ IGFBP2,^[44]^ IGFBP5,^[45]^ IL6,^[46]^ POSTN,^[47]^ RARRES2,^[48]^ SERPINE1,^[49]^ TIMP1,^[50]^ genetic polymorphisms of EGF,^[51]^ and epigenetic variations of both CXCL2 and CXCL5^[52]^ were shown to underlie PAC pathogenesis and prognosis. Functional enrichment and GCN analyses for the LGI3-regulated genes that were altered in NSCLC indicated their involvement in similar GO categories.^[18]^ By contrast, LGI3-regulated gene products that were altered in glioma cases were predominantly associated with hypoxia, cell proliferation, angiogenesis, p53, and HIF-1 pathways.^[17]^ Therefore, these results show that LGI3-regulated gene products may play pathological roles in PAC and other cancers through overlapping and distinctive mechanisms.

A number of gene products that were changed in metastatic PAC compared with the primary tumor may account for the components that form the PAC microenvironment. These components can include angiogenesis, lymphangiogenesis, desmoplasia, epithelial-mesenchymal transition, migration, invasion, and pre-metastatic niche formation.^[53–55]^ Five of the gene products from among 13 metastasis-specific PAC-altered, LGI3-regulated genes (AHSG, CCL11, CXCL13, GAS6, and TNFSF13B; Fig. 4B, gray nodes) were shown to be either cytokines, chemokines, or other secreted factors. Involvement in tumor metastasis was postulated for AHSG in head and neck squamous cell carcinoma,^[56]^ CCL11 and CXCL13 in lung cancer,^[57,58]^ GAS6 in PAC,^[59]^ and TNFSF13B in glioma^[60]^ Therefore, the dysregulation of LGI3 in PAC may affect PAC metastasis through intercellular communication in the tumor microenvironment.

The functional signatures of LGI3-regulated, PAC-altered gene products in the PAC regulon, which is a transcriptional regulatory network of PAC, provided an insight into their involvement with the cellular processes that underlie complex pathologic phenotypes (Fig. 5)^[30,61]^ One particular subset of the PAC regulon (Group b, Fig. 5) was directly associated with LGI3-regulated, PAC-altered gene products, and the GO categories of the genes in group b (Fig. 5) were similar to the results from a GCN analysis of a healthy pancreas (Fig. 3). Overall, the transcriptional regulatory network of LGI3-regulated gene products involved in inflammatory and immune responses, metabolic regulation, cell differentiation, and angiogenesis in a healthy pancreas may be dysregulated in PAC, which could account for their significant effect on PAC pathogenesis.

The correlation of low LGI3 expression with poor PAC prognosis (Fig. 6) suggested that LGI3 may be able to suppress progression of PAC. Notably, a significant reduction in LGI3 expression in stage II PAC (Fig. 6C) suggested its role in the early stage of metastasis. Furthermore, the functional network of LGI3-regulated and PAC-prognostic genes (Fig. 7B) highlighted the crucial role of well-known prognostic factors of PAC, such as EGFR tyrosine kinase, PD-1 checkpoint pathway, and inflammatory cytokine functions.^[62–64]^ LGI3 has been shown to regulate signaling proteins and transcription factors involved in various cancers including Akt, β-catenin, focal adhesion kinase, MDM2, MITF, NF-κB and p53.^[3,4,10,13,65–67]^ Perturbed regulation of these proteins by decreased LGI3 levels in PAC may account for prognostic mechanisms of LGI3 in PAC as well as in other cancers.^[14]^

It has been postulated that LGI3 may be part of the cytokine network in obesity-related metabolic diseases and cancers.^[14]^ Cytokine networks play critical roles in cancer-immune interactions in the PAC microenvironment,^[53,54]^ while the upregulation of LGI3 in the plasma and adipose tissues of obese individuals was postulated to promote chronic inflammation and cancers.^[11,12]^ Cytokine perturbation in obese individuals may increase the risk of PAC as well as cancers of the liver, the gastrointestinal tract, and reproductive organs.^[68–70]^ This seems to indicate that the presence of LGI3-regulated adipokine networks in obese individuals may highlight a link between obesity and PAC.^[11,13]^

Tumor-associated macrophages (TAMs) are one of the most abundant immune cell populations found in the PAC microenvironment and the deviation of TAM polarization from the M2 to the M1 type with anti-tumorigenic activities correlated with positive PAC prognosis.^[55,71,72]^ LGI3 was shown to increase M1-polarized macrophage markers (TNF-α, iNOS, CCL-2/MCP-1, IL-6),^[12–14]^ while NF-κB was shown to be a key transcription factor in the mutual upregulation of LGI3 and TNF-α.^[13]^ It can therefore be speculated that LGI3 may contribute to anti-tumor effects in the PAC microenvironment by promoting and maintaining the M1-polarization of TAMs. Additionally, the downregulation of LGI3 in PAC may play a role in the perturbation of TAM-related immune and inflammatory cytokine networks in the pancreas and may also account for the prognostic mechanisms of PAC. Furthermore, LGI3 expression and its negative correlation with infiltration of tumor immune suppressor cells and its positive correlation with infiltration of tumor immune promoters or modulators (macrophages, monocytes, mDC, CD4 + T cells, CD8 + T cells, natural killer cells and memory B cells)^[54]^ (Fig. 8) suggested that LGI3 is a tumor-suppressing cytokine that regulates immune cell infiltration in PAC microenvironment.

## 5. Conclusion

In conclusion, these results provide an integrative insight into the prognostic value of LGI3 in PAC by demonstrating the regulatory networks of LGI3-regulated and PAC-altered gene products and the prognostic association of LGI3 expression in PAC cases. This study proposes, therefore, that LGI3 plays both a pathological and a prognostic role in PAC progression by influencing cytokine network in the tumor microenvironment.

## Author contribution

**Conceptualization:** Hye-Young Yun.

**Data curation:** Hye-Young Yun.

**Formal analysis:** Hye-Young Yun.

**Funding acquisition:** Hye-Young Yun.

**Investigation:** Hye-Young Yun.

**Methodology:** Hye-Young Yun.

**Project administration:** Hye-Young Yun.

**Resources:** Hye-Young Yun.

**Software:** Hye-Young Yun.

**Supervision:** Hye-Young Yun.

**Validation:** Hye-Young Yun.

**Visualization:** Hye-Young Yun.

**Writing – original draft:** Hye-Young Yun.

**Writing – review & editing:** Hye-Young Yun.

## Supplementary Material
















