# Supplementary material for: Leucine rich repeat LGI family member 3: Integrative analyses support its prognostic association with pancreatic adenocarcinoma
Source: Medicine (Baltimore). 2024 Feb 23;103(8):e37183. doi: 10.1097/MD.0000000000037183 (PMC11309673; doi:10.1097/MD.0000000000037183)
Supplement: Supplementary file 1 [file medi-103-e37183-s001.docx]

Table S1. Clinicopathological features of the TCGA and ICGC cohorts.

| Variables |  | TCGA | ICGC |
| --- | --- | --- | --- |
| Gender |  |  |  |
|  | Male | 97 | 137 |
|  | Female | 80 | 120 |
| Age |  |  |  |
|  |  | Median (range)  65 (36–89) | <65 103  ≥65 154 |
| Histological type |  |  |  |
|  | PDAC | 164 | 257 |
|  | IPMN | NA | 0 |
|  | Neuroendocrine | 6 | 0 |
|  | Others | 7 | 0 |
| Location |  |  |  |
|  | Head | 129 | NA |
|  | Body | 15 | NA |
|  | Tail | 14 | NA |
|  | Others | 19 | NA |
| T stage |  |  |  |
|  | T1 | 7 | NA |
|  | T2 | 24 | NA |
|  | T3 | 141 | NA |
|  | T4 | 3 | NA |
|  | Others | 2 | NA |
| N stage |  |  |  |
|  | N0 | 49 | NA |
|  | N1 | 119 | NA |
|  | Others | 9 | NA |
| AJCC stage |  |  |  |
|  | I | 21 | NA |
|  | IIA | 28 | NA |
|  | IIB | 118 | NA |
|  | III | 3 | NA |
|  | IV | 4 | NA |
|  | Others | 3 | NA |
| Margin status |  |  |  |
|  | R0 | 83 | NA |
|  | R1 | 41 | NA |
|  | Others | 53 | NA |
