# Supplementary material for: Leucine rich repeat LGI family member 3: Integrative analyses support its prognostic association with pancreatic adenocarcinoma
Source: Medicine (Baltimore). 2024 Feb 23;103(8):e37183. doi: 10.1097/MD.0000000000037183 (PMC11309673; doi:10.1097/MD.0000000000037183)
Supplement: Supplementary file 2 [file medi-103-e37183-s002.docx]

Table S2. List of genes that showed altered expression in PAC tissues.

| Groups | Gene name |
| --- | --- |
| Upregulated | A1CF |
|  | A2M |
|  | ABCA10 |
|  | ABCA5 |
|  | ABCA6 |
|  | ABCA8 |
|  | ABCA9 |
|  | ABCB1 |
|  | ABCC2 |
|  | ABCG5 |
|  | ACACB |
|  | ACADL |
|  | ACADM |
|  | ACAT1 |
|  | ACRBP |
|  | ACSM2A |
|  | ACSM3 |
|  | ACSS1 |
|  | ACVR1C |
|  | ADAD2 |
|  | ADAMTS1 |
|  | ADAMTS7 |
|  | ADAMTSL3 |
|  | ADARB2 |
|  | ADCY1 |
|  | ADGRV1 |
|  | ADH1A |
|  | ADH1B |
|  | ADH1C |
|  | ADH4 |
|  | ADHFE1 |
|  | ADIPOQ |
|  | ADRA2B |
|  | AFAP1L1 |
|  | AGER |
|  | AGXT2 |
|  | AKAP7 |
|  | ALDH1A1 |
|  | ALDH1L2 |
|  | ALDH3A2 |
|  | ALDH6A1 |
|  | ALKAL2 |
|  | AMY1B |
|  | AMY1C |
|  | AMY2A |
|  | AMY2B |
|  | ANGPTL1 |
|  | ANGPTL6 |
|  | ANKEF1 |
|  | ANKRD2 |
|  | ANKRD20A1 |
|  | ANKRD20A21P |
|  | ANKRD20A2P |
|  | ANKRD20A3P |
|  | ANKRD20A4P |
|  | ANKRD20A5P |
|  | ANKRD20A8P |
|  | ANKRD20A9P |
|  | ANKRD62 |
|  | ANO5 |
|  | ANPEP |
|  | AOPEP |
|  | APLP1 |
|  | APOA1 |
|  | APOA4 |
|  | APOB |
|  | APOBEC2 |
|  | APOC2 |
|  | APOC3 |
|  | APOH |
|  | APOL5 |
|  | APOLD1 |
|  | APOM |
|  | AQP1 |
|  | AQP12A |
|  | AQP12B |
|  | AQP7 |
|  | ARHGAP24 |
|  | ARHGAP30 |
|  | ARHGDIG |
|  | ARSL |
|  | ART3 |
|  | AS3MT |
|  | ASB2 |
|  | ASB9 |
|  | ASPA |
|  | ATAD3C |
|  | ATF5 |
|  | ATP13A4 |
|  | ATP2A3 |
|  | ATP4A |
|  | ATP8A1 |
|  | ATRNL1 |
|  | AVPR1A |
|  | AZGP1 |
|  | B3GALNT2 |
|  | BACE1 |
|  | BANF2 |
|  | BCAM |
|  | BCAT1 |
|  | BCL11A |
|  | BCL2 |
|  | BEX2 |
|  | BEX5 |
|  | BMP5 |
|  | BORCS7-ASMT |
|  | BRSK2 |
|  | BTG2 |
|  | BTNL9 |
|  | C1orf115 |
|  | C20orf144 |
|  | C21orf58 |
|  | C2CD4B |
|  | C5 |
|  | C6 |
|  | C7 |
|  | C8G |
|  | C9orf131 |
|  | CA4 |
|  | CACHD1 |
|  | CACNA1E |
|  | CACNA1I |
|  | CACNB2 |
|  | CADM3 |
|  | CADPS |
|  | CAMK2B |
|  | CAND2 |
|  | CASR |
|  | CASTOR2 |
|  | CASTOR3P |
|  | CATSPERB |
|  | CBFA2T3 |
|  | CBS |
|  | CCDC110 |
|  | CCDC141 |
|  | CCDC198 |
|  | CCDC69 |
|  | CCL2 |
|  | CD209 |
|  | CD302 |
|  | CD36 |
|  | CD5L |
|  | CD79A |
|  | CDH15 |
|  | CDH22 |
|  | CEBPE |
|  | CELA2A |
|  | CELA3A |
|  | CELA3B |
|  | CELP |
|  | CERKL |
|  | CERS4 |
|  | CETP |
|  | CFAP221 |
|  | CFAP70 |
|  | CFAP91 |
|  | CFD |
|  | CFTR |
|  | CGNL1 |
|  | CHAC1 |
|  | CHAD |
|  | CHGA |
|  | CHN2 |
|  | CHODL |
|  | CHRDL1 |
|  | CHRM3 |
|  | CHRNA4 |
|  | CHST13 |
|  | CHST9 |
|  | CIDEB |
|  | CIRBP |
|  | CITED2 |
|  | CKM |
|  | CLDN10 |
|  | CLEC4G |
|  | CLEC4M |
|  | CLIC5 |
|  | CLTRN |
|  | CLU |
|  | CMTM8 |
|  | CNKSR3 |
|  | CNTFR |
|  | COBLL1 |
|  | COCH |
|  | COQ8A |
|  | COX6A2 |
|  | CPA1 |
|  | CPEB3 |
|  | CPLX3 |
|  | CRAT |
|  | CREB3L3 |
|  | CRELD2 |
|  | CRYAB |
|  | CTH |
|  | CTRB2 |
|  | CTTNBP2 |
|  | CXCL12 |
|  | CXCL2 |
|  | CXXC4 |
|  | CYB561D1 |
|  | CYB5A |
|  | CYP1A2 |
|  | CYP26B1 |
|  | CYP2D6 |
|  | CYP2E1 |
|  | CYP39A1 |
|  | CYP4A11 |
|  | CYP4F2 |
|  | CYP4V2 |
|  | CYYR1 |
|  | DAO |
|  | DAPL1 |
|  | DBH |
|  | DCDC2 |
|  | DDC |
|  | DEFB1 |
|  | DES |
|  | DIRAS3 |
|  | DLK1 |
|  | DNAH14 |
|  | DNAJB9 |
|  | DNAJC12 |
|  | DNASE1 |
|  | DNASE1L3 |
|  | DPEP1 |
|  | DPF3 |
|  | DPP10 |
|  | DPT |
|  | DTNA |
|  | DUSP1 |
|  | DUSP13 |
|  | ECHDC3 |
|  | ECI2 |
|  | EDN3 |
|  | EDNRB |
|  | EGF |
|  | ELAC1 |
|  | ELAPOR1 |
|  | ELOVL2 |
|  | EMCN |
|  | EML5 |
|  | ENAM |
|  | ENDOG |
|  | ENPP1 |
|  | ENPP5 |
|  | ENTPD3 |
|  | EPB41L5 |
|  | EPB42 |
|  | EPHX1 |
|  | EPHX2 |
|  | EPO |
|  | ERBB4 |
|  | ESRRG |
|  | EVX1 |
|  | F10 |
|  | F2 |
|  | F8 |
|  | FABP1 |
|  | FABP4 |
|  | FAM107A |
|  | FAM149A |
|  | FAM171A1 |
|  | FAM24B-CUZD1 |
|  | FAM30A |
|  | FAM3B |
|  | FAM43A |
|  | FAM47E |
|  | FAM47E-STBD1 |
|  | FBLN5 |
|  | FBP2 |
|  | FBXW12 |
|  | FCN2 |
|  | FGF10 |
|  | FGF12 |
|  | FGFR1 |
|  | FHL5 |
|  | FICD |
|  | FITM1 |
|  | FKBP11 |
|  | FLRT2 |
|  | FLT3 |
|  | FOSB |
|  | FOXD3 |
|  | FOXN3 |
|  | FOXP2 |
|  | FREM1 |
|  | FTCD |
|  | FXYD2 |
|  | FXYD6-FXYD2 |
|  | FYCO1 |
|  | FZD3 |
|  | FZD9 |
|  | G6PC2 |
|  | GAD2 |
|  | GALR3 |
|  | GAS2 |
|  | GATA4 |
|  | GBA3 |
|  | GC |
|  | GCAT |
|  | GCGR |
|  | GCNT4 |
|  | GCOM1 |
|  | GDAP1 |
|  | GDF10 |
|  | GGTLC2 |
|  | GHR |
|  | GHRL |
|  | GIPC2 |
|  | GKAP1 |
|  | GLS2 |
|  | GLTPD2 |
|  | GMNN |
|  | GNMT |
|  | GOLGA8A |
|  | GP2 |
|  | GPAM |
|  | GPC3 |
|  | GPD1 |
|  | GPHA2 |
|  | GPR155 |
|  | GPR50 |
|  | GPR78 |
|  | GPT |
|  | GPT2 |
|  | GPX3 |
|  | GRAMD1C |
|  | GRB10 |
|  | GRB14 |
|  | GRIN1 |
|  | GRK5 |
|  | GRPR |
|  | GSTA1 |
|  | GSTA2 |
|  | GSTA3 |
|  | GSTA4 |
|  | GSTM1 |
|  | GUCA1C |
|  | HABP2 |
|  | HADH |
|  | HBA1 |
|  | HBA2 |
|  | HBB |
|  | HBD |
|  | HBQ1 |
|  | HDHD2 |
|  | HEPACAM2 |
|  | HGD |
|  | HGFAC |
|  | HHEX |
|  | HID1 |
|  | HIPK2 |
|  | HLF |
|  | HMGCLL1 |
|  | HMGCS2 |
|  | HNF1A-AS1 |
|  | HNF1B |
|  | HOMER2 |
|  | HOOK1 |
|  | HPN |
|  | HSPA12B |
|  | HSPA1L |
|  | HSPA4L |
|  | HSPB8 |
|  | HSPD1 |
|  | HUNK |
|  | IAPP |
|  | IDH2 |
|  | IFRD1 |
|  | IFT20 |
|  | IGF1 |
|  | IGFALS |
|  | IGFBP2 |
|  | IGFN1 |
|  | IGHG1 |
|  | IL11RA |
|  | IL12RB2 |
|  | IL1B |
|  | IL1RL1 |
|  | IL22RA1 |
|  | IL6 |
|  | IMPA2 |
|  | INSIG1 |
|  | INSL3 |
|  | IRAG2 |
|  | ITGA8 |
|  | ITIH4 |
|  | ITLN1 |
|  | JADE1 |
|  | KANK3 |
|  | KAT2B |
|  | KCNJ3 |
|  | KCNJ5 |
|  | KCNJ8 |
|  | KCNMB2 |
|  | KCTD16 |
|  | KHK |
|  | KIF12 |
|  | KIF1A |
|  | KIRREL2 |
|  | KLB |
|  | KLF14 |
|  | KLF15 |
|  | KLF9 |
|  | KLHL13 |
|  | KLK1 |
|  | KLK3 |
|  | KLKB1 |
|  | KRT81 |
|  | KRT9 |
|  | KSR1 |
|  | L3MBTL4 |
|  | LARP1B |
|  | LDB3 |
|  | LEAP2 |
|  | LGALS2 |
|  | LIFR |
|  | LINC00339 |
|  | LINC00643 |
|  | LINC00671 |
|  | LINC02905 |
|  | LMO3 |
|  | LNX1 |
|  | LOC102724560 |
|  | LOC285097 |
|  | LONRF2 |
|  | LPA |
|  | LPAR3 |
|  | LRIG1 |
|  | LTB4R2 |
|  | LYVE1 |
|  | MAL |
|  | MAN1A1 |
|  | MAOA |
|  | MAP3K21 |
|  | MAPK8IP1 |
|  | MASP1 |
|  | MASP2 |
|  | MAT1A |
|  | MCOLN3 |
|  | MEIS1 |
|  | MESP1 |
|  | METTL7A |
|  | MFNG |
|  | MGAM2 |
|  | MGAT4A |
|  | MIR29B2CHG |
|  | MKNK1 |
|  | MKNK2 |
|  | MLC1 |
|  | MLXIPL |
|  | MLYCD |
|  | MMP17 |
|  | MMP25 |
|  | MMRN1 |
|  | MOAP1 |
|  | MPP7 |
|  | MPV17L |
|  | MRO |
|  | MSRA |
|  | MSRB1 |
|  | MT1A |
|  | MT1E |
|  | MT1F |
|  | MT1G |
|  | MT1H |
|  | MT1L |
|  | MT1M |
|  | MT1X |
|  | MT2A |
|  | MT3 |
|  | MTERF2 |
|  | MTTP |
|  | MTUS2 |
|  | MUC15 |
|  | MUC6 |
|  | MYBPC2 |
|  | MYBPH |
|  | MYC |
|  | MYEF2 |
|  | MYH11 |
|  | MYH7 |
|  | MYLK2 |
|  | MYO3A |
|  | MYOC |
|  | MYOM1 |
|  | MYOM2 |
|  | MYRIP |
|  | MYZAP |
|  | NAA16 |
|  | NAP1L4 |
|  | NCDN |
|  | NCR2 |
|  | NDUFV3 |
|  | NEU3 |
|  | NEURL1 |
|  | NEUROD1 |
|  | NFIB |
|  | NIBAN1 |
|  | NMNAT2 |
|  | NOS2 |
|  | NOSIP |
|  | NOSTRIN |
|  | NPHS1 |
|  | NPY1R |
|  | NR1I3 |
|  | NR3C2 |
|  | NR4A1 |
|  | NR4A2 |
|  | NR4A3 |
|  | NR5A2 |
|  | NRCAM |
|  | NRG4 |
|  | NRN1 |
|  | NRXN3 |
|  | NTRK2 |
|  | NUDT7 |
|  | NUPR1 |
|  | NUTF2 |
|  | ONECUT1 |
|  | OR10G4 |
|  | OR10G7 |
|  | OR10G8 |
|  | OR10G9 |
|  | OR4D5 |
|  | OR8D4 |
|  | OTC |
|  | P2RX1 |
|  | PABPC4 |
|  | PACRG |
|  | PAH |
|  | PAIP2B |
|  | PAK3 |
|  | PALS2 |
|  | PAPPA2 |
|  | PARM1 |
|  | PARP10 |
|  | PAX6 |
|  | PBLD |
|  | PBX1 |
|  | PCDHB5 |
|  | PCSK1 |
|  | PCSK1N |
|  | PCSK2 |
|  | PDCD4 |
|  | PDE3B |
|  | PDE5A |
|  | PDE8B |
|  | PDGFD |
|  | PDK2 |
|  | PDK4 |
|  | PDZK1 |
|  | PDZK1P1 |
|  | PEBP4 |
|  | PELI2 |
|  | PEX5L |
|  | PGA3 |
|  | PGA5 |
|  | PGAM2 |
|  | PGLYRP1 |
|  | PHGDH |
|  | PHYHD1 |
|  | PHYHIPL |
|  | PI16 |
|  | PIR-FIGF |
|  | PKHD1 |
|  | PKNOX2 |
|  | PLA2G12A |
|  | PLA2G12B |
|  | PLA2G1B |
|  | PLA2G2A |
|  | PLA2G5 |
|  | PLAAT5 |
|  | PLCB1 |
|  | PLIN1 |
|  | PLIN4 |
|  | PLTP |
|  | PM20D1 |
|  | POMC |
|  | PON3 |
|  | PPP1R1A |
|  | PRDX4 |
|  | PRKAR2B |
|  | PRLR |
|  | PRODH2 |
|  | PROX1 |
|  | PRPH2 |
|  | PSAT1 |
|  | PSMD6 |
|  | PTGER4 |
|  | PTP4A3 |
|  | PTPRN2 |
|  | PTX3 |
|  | PWAR6 |
|  | PWWP3B |
|  | PXMP2 |
|  | PYY2 |
|  | QPRT |
|  | RAB26 |
|  | RAB39B |
|  | RAB3C |
|  | RAB3D |
|  | RADIL |
|  | RAP1GAP |
|  | RAP1GAP2 |
|  | RARRES2 |
|  | RASD1 |
|  | RASGRP2 |
|  | RASL10B |
|  | RBP1 |
|  | RBP4 |
|  | RBPJL |
|  | RCOR2 |
|  | REEP1 |
|  | REG1B |
|  | REG1CP |
|  | REG3A |
|  | REG3G |
|  | RETREG1 |
|  | RFX6 |
|  | RGN |
|  | RGS2 |
|  | RIC3 |
|  | RILP |
|  | RNASE1 |
|  | RNF186 |
|  | RNF212 |
|  | ROBO4 |
|  | ROPN1L |
|  | RPH3AL |
|  | RPL3L |
|  | RPS4Y1 |
|  | RSPO3 |
|  | RTP3 |
|  | RYR2 |
|  | S100A5 |
|  | S100A8 |
|  | S1PR4 |
|  | SAT2 |
|  | SCAMP5 |
|  | SCD5 |
|  | SCG3 |
|  | SCGB3A2 |
|  | SCGN |
|  | SCTR |
|  | SDF2L1 |
|  | SDK1 |
|  | SEC11C |
|  | SEL1L |
|  | SELE |
|  | SELENOP |
|  | SELP |
|  | SEMA6A |
|  | SEMA6D |
|  | SERPINA3 |
|  | SERPINA4 |
|  | SERPINA5 |
|  | SERPINA6 |
|  | SERPINI1 |
|  | SETBP1 |
|  | SFRP1 |
|  | SFRP5 |
|  | SFTPC |
|  | SFTPD |
|  | SGK1 |
|  | SH3BGR |
|  | SHBG |
|  | SHISA4 |
|  | SHISAL2B |
|  | SIDT2 |
|  | SIGLEC11 |
|  | SLAIN1 |
|  | SLC16A10 |
|  | SLC16A12 |
|  | SLC16A7 |
|  | SLC17A4 |
|  | SLC1A2 |
|  | SLC22A7 |
|  | SLC25A15 |
|  | SLC25A20 |
|  | SLC26A1 |
|  | SLC27A5 |
|  | SLC28A1 |
|  | SLC2A2 |
|  | SLC30A2 |
|  | SLC30A8 |
|  | SLC35G1 |
|  | SLC38A3 |
|  | SLC38A4 |
|  | SLC38A5 |
|  | SLC39A14 |
|  | SLC39A5 |
|  | SLC39A8 |
|  | SLC3A1 |
|  | SLC41A1 |
|  | SLC43A1 |
|  | SLC4A4 |
|  | SLC6A16 |
|  | SLC7A2 |
|  | SLIT3 |
|  | SLURP1 |
|  | SMPD1 |
|  | SMPDL3B |
|  | SMYD1 |
|  | SNORD114-3 |
|  | SNTG2 |
|  | SNURF |
|  | SOCS3 |
|  | SORBS2 |
|  | SOX1 |
|  | SPINK1 |
|  | SPP2 |
|  | SPX |
|  | SRPX |
|  | SSR4 |
|  | SST |
|  | ST8SIA3 |
|  | STAB2 |
|  | STEAP4 |
|  | STK33 |
|  | STXBP6 |
|  | SULT2A1 |
|  | SV2B |
|  | SYBU |
|  | SYCN |
|  | SYNPO2 |
|  | SYT4 |
|  | TBC1D30 |
|  | TBX1 |
|  | TC2N |
|  | TCAP |
|  | TCEA3 |
|  | TCEAL2 |
|  | TCF15 |
|  | TDH |
|  | TENT5C |
|  | TEX11 |
|  | TF |
|  | TFPI2 |
|  | TGFBR3 |
|  | THRSP |
|  | TIFA |
|  | TKTL1 |
|  | TLCD4 |
|  | TLCD4-RWDD3 |
|  | TM4SF18 |
|  | TMEM131L |
|  | TMEM220 |
|  | TMEM51-AS1 |
|  | TMEM52 |
|  | TMEM88 |
|  | TMEM97 |
|  | TMOD1 |
|  | TMPRSS6 |
|  | TNFRSF10C |
|  | TNNT3 |
|  | TNXA |
|  | TNXB |
|  | TP53INP1 |
|  | TPD52L1 |
|  | TPO |
|  | TPSAB1 |
|  | TPSG1 |
|  | TRHDE |
|  | TRPV6 |
|  | TSGA13 |
|  | TSPAN7 |
|  | TTLL7 |
|  | TTN |
|  | TTTY14 |
|  | TUSC3 |
|  | UGT2A3 |
|  | UGT2B10 |
|  | UGT2B11 |
|  | UGT2B15 |
|  | UGT2B17 |
|  | UGT2B7 |
|  | UNC79 |
|  | UPB1 |
|  | USP2 |
|  | USP44 |
|  | UTS2R |
|  | VAV3 |
|  | VEGFD |
|  | VEPH1 |
|  | VIPR2 |
|  | VLDLR |
|  | VTCN1 |
|  | WDR17 |
|  | WNK2 |
|  | WNT6 |
|  | XBP1 |
|  | YIPF2 |
|  | ZBED3 |
|  | ZBTB16 |
|  | ZDBF2 |
|  | ZNF189 |
|  | ZNF33B |
|  | ZNF704 |
|  | ZNF737 |
|  | ZNF784 |
|  | ZSCAN10 |
| Downregulated | A2ML1 |
|  | ABCA1 |
|  | ABCA12 |
|  | ABCC3 |
|  | ABCG1 |
|  | ABLIM3 |
|  | ABR |
|  | ACP5 |
|  | ACSL5 |
|  | ACTA2 |
|  | ACTB |
|  | ACTG1 |
|  | ACTG2 |
|  | ACTN1 |
|  | ACTN4 |
|  | ACTR3 |
|  | ACVR1 |
|  | ACVR2A |
|  | ADAM10 |
|  | ADAM12 |
|  | ADAM19 |
|  | ADAM22 |
|  | ADAM28 |
|  | ADAM8 |
|  | ADAM9 |
|  | ADAMTS12 |
|  | ADAMTS2 |
|  | ADAMTS6 |
|  | ADAP1 |
|  | ADGRE5 |
|  | ADGRF1 |
|  | ADGRG6 |
|  | ADM |
|  | ADO |
|  | ADORA2B |
|  | ADTRP |
|  | AEBP1 |
|  | AFAP1 |
|  | AFAP1-AS1 |
|  | AGMO |
|  | AGR2 |
|  | AGR3 |
|  | AGRN |
|  | AHNAK |
|  | AHNAK2 |
|  | AHR |
|  | AK4 |
|  | AKR1B10 |
|  | ALDOA |
|  | ALDOC |
|  | ALOX5 |
|  | ALOX5AP |
|  | AMIGO2 |
|  | AMPD3 |
|  | ANGPT2 |
|  | ANGPTL2 |
|  | ANKH |
|  | ANKLE2 |
|  | ANKRD1 |
|  | ANKRD10 |
|  | ANKRD22 |
|  | ANKRD36B |
|  | ANKRD50 |
|  | ANLN |
|  | ANO1 |
|  | ANOS1 |
|  | ANTXR1 |
|  | ANTXR2 |
|  | ANXA1 |
|  | ANXA10 |
|  | ANXA2 |
|  | ANXA2P1 |
|  | ANXA2P2 |
|  | ANXA3 |
|  | ANXA4 |
|  | ANXA8 |
|  | ANXA8L1 |
|  | AP1S3 |
|  | AP2B1 |
|  | AP3M2 |
|  | APBB2 |
|  | APCDD1 |
|  | APLP2 |
|  | APOL1 |
|  | APOL2 |
|  | AQP9 |
|  | ARAP2 |
|  | ARAP3 |
|  | AREG |
|  | ARF4 |
|  | ARGLU1 |
|  | ARHGAP1 |
|  | ARHGAP11A |
|  | ARHGAP21 |
|  | ARHGAP26 |
|  | ARHGAP42 |
|  | ARHGAP5 |
|  | ARHGDIB |
|  | ARL14 |
|  | ARL4C |
|  | ARL6IP1 |
|  | ARMC9 |
|  | ARNT2 |
|  | ARNTL2 |
|  | ARPC1B |
|  | ARPC2 |
|  | ASAP2 |
|  | ASPH |
|  | ASPHD1 |
|  | ASPHD2 |
|  | ASPM |
|  | ASPN |
|  | ATAD2 |
|  | ATL1 |
|  | ATP10A |
|  | ATP10B |
|  | ATP1B3 |
|  | ATP2B4 |
|  | ATP2C2 |
|  | ATP6AP1L |
|  | ATP7A |
|  | AURKA |
|  | AXL |
|  | B2M |
|  | B3GALT5 |
|  | B3GNT3 |
|  | B3GNT5 |
|  | B4GALT4 |
|  | B4GALT5 |
|  | BACE2 |
|  | BAIAP2L1 |
|  | BANK1 |
|  | BCAS1 |
|  | BCL10 |
|  | BCL2L1 |
|  | BCL2L15 |
|  | BGN |
|  | BHLHE40 |
|  | BHLHE41 |
|  | BICD1 |
|  | BIK |
|  | BIRC3 |
|  | BMP1 |
|  | BMP4 |
|  | BMPR2 |
|  | BNC2 |
|  | BPIFB1 |
|  | BRIP1 |
|  | BST2 |
|  | BTBD10 |
|  | BUB1 |
|  | BUB1B |
|  | BZW1 |
|  | C11orf80 |
|  | C15orf48 |
|  | C19orf33 |
|  | C1GALT1 |
|  | C1orf116 |
|  | C1QTNF3 |
|  | C1QTNF3-AMACR |
|  | C1QTNF5 |
|  | C1R |
|  | C1S |
|  | C3orf14 |
|  | C4BPB |
|  | C4orf48 |
|  | C5orf15 |
|  | C5orf46 |
|  | CA13 |
|  | CA9 |
|  | CACNA1C |
|  | CACNB3 |
|  | CALD1 |
|  | CALHM5 |
|  | CALM2 |
|  | CALML4 |
|  | CALU |
|  | CAMK2N1 |
|  | CAP1 |
|  | CAPG |
|  | CAPN1 |
|  | CAPN2 |
|  | CAPN8 |
|  | CAPN9 |
|  | CAPRIN2 |
|  | CAPZB |
|  | CARD10 |
|  | CARD11 |
|  | CASK |
|  | CAST |
|  | CAVIN1 |
|  | CCDC14 |
|  | CCDC80 |
|  | CCL13 |
|  | CCL18 |
|  | CCL20 |
|  | CCN4 |
|  | CCNA2 |
|  | CCNB1 |
|  | CCNB2 |
|  | CCND1 |
|  | CCR7 |
|  | CD109 |
|  | CD1E |
|  | CD200 |
|  | CD248 |
|  | CD47 |
|  | CD53 |
|  | CD55 |
|  | CD58 |
|  | CD68 |
|  | CD74 |
|  | CD80 |
|  | CD82 |
|  | CD86 |
|  | CD9 |
|  | CDA |
|  | CDC6 |
|  | CDCA7 |
|  | CDCP1 |
|  | CDH1 |
|  | CDH11 |
|  | CDH13 |
|  | CDH17 |
|  | CDH2 |
|  | CDH3 |
|  | CDHR2 |
|  | CDK1 |
|  | CDK6 |
|  | CDKL5 |
|  | CDKN2A |
|  | CDKN2B |
|  | CDR2L |
|  | CDS1 |
|  | CEACAM1 |
|  | CEACAM5 |
|  | CEACAM6 |
|  | CEACAM7 |
|  | CELSR1 |
|  | CEMIP |
|  | CENPF |
|  | CENPI |
|  | CENPK |
|  | CEP170 |
|  | CEP55 |
|  | CERS6 |
|  | CETN2 |
|  | CFH |
|  | CFL1 |
|  | CGAS |
|  | CGN |
|  | CHEK1 |
|  | CHML |
|  | CHMP2B |
|  | CHST11 |
|  | CILP |
|  | CIP2A |
|  | CIT |
|  | CKAP2 |
|  | CKLF |
|  | CKLF-CMTM1 |
|  | CKMT1A |
|  | CKMT1B |
|  | CKS1B |
|  | CKS2 |
|  | CLDN1 |
|  | CLDN12 |
|  | CLDN18 |
|  | CLDN23 |
|  | CLDN4 |
|  | CLDN7 |
|  | CLEC4A |
|  | CLEC5A |
|  | CLEC7A |
|  | CLIC1 |
|  | CLIC3 |
|  | CLIC6 |
|  | CLIP4 |
|  | CLMP |
|  | CLRN3 |
|  | CLTB |
|  | CMTM1 |
|  | CMTM3 |
|  | CMTM6 |
|  | CMTM7 |
|  | CNIH4 |
|  | CNN1 |
|  | CNN2 |
|  | COL10A1 |
|  | COL11A1 |
|  | COL12A1 |
|  | COL13A1 |
|  | COL15A1 |
|  | COL16A1 |
|  | COL17A1 |
|  | COL1A1 |
|  | COL1A2 |
|  | COL3A1 |
|  | COL4A1 |
|  | COL4A2 |
|  | COL5A1 |
|  | COL5A2 |
|  | COL6A3 |
|  | COL7A1 |
|  | COL8A1 |
|  | COL8A2 |
|  | COLEC12 |
|  | COLGALT1 |
|  | COMP |
|  | CORIN |
|  | CORO1C |
|  | CORO2A |
|  | CP |
|  | CPE |
|  | CPSF3 |
|  | CRABP2 |
|  | CRIP1 |
|  | CRISPLD1 |
|  | CSGALNACT2 |
|  | CST1 |
|  | CST2 |
|  | CST6 |
|  | CSTB |
|  | CTDSPL |
|  | CTHRC1 |
|  | CTLA4 |
|  | CTNNA1 |
|  | CTNNB1 |
|  | CTSA |
|  | CTSB |
|  | CTSC |
|  | CTSD |
|  | CTSE |
|  | CTSK |
|  | CTSS |
|  | CTSV |
|  | CTSZ |
|  | CTTN |
|  | CTTNBP2NL |
|  | CXCL10 |
|  | CXCL14 |
|  | CXCL16 |
|  | CXCL5 |
|  | CXCL8 |
|  | CXCR4 |
|  | CXorf38 |
|  | CYB5B |
|  | CYB5R2 |
|  | CYBB |
|  | CYP1B1 |
|  | CYP24A1 |
|  | CYP2C18 |
|  | CYP2S1 |
|  | CYP3A5 |
|  | CYTH3 |
|  | CYTOR |
|  | DACT1 |
|  | DAPP1 |
|  | DBN1 |
|  | DCBLD1 |
|  | DCBLD2 |
|  | DDR1 |
|  | DDR2 |
|  | DDX5 |
|  | DDX58 |
|  | DDX60 |
|  | DDX60L |
|  | DEPDC1B |
|  | DGKA |
|  | DGKH |
|  | DHCR24 |
|  | DHFR |
|  | DHRS9 |
|  | DIO2 |
|  | DIP2B |
|  | DKK1 |
|  | DKK3 |
|  | DLG5 |
|  | DLGAP5 |
|  | DMBT1 |
|  | DNAJC15 |
|  | DNAJC9 |
|  | DNM3 |
|  | DNTTIP1 |
|  | DOCK11 |
|  | DOCK5 |
|  | DOK4 |
|  | DOK5 |
|  | DPY19L1 |
|  | DPYSL3 |
|  | DSC2 |
|  | DSC3 |
|  | DSE |
|  | DSG2 |
|  | DSG3 |
|  | DTL |
|  | DTX3L |
|  | DUOX2 |
|  | DUSP10 |
|  | DUSP4 |
|  | DUSP5 |
|  | DUSP6 |
|  | DYNLT1 |
|  | DYRK2 |
|  | E2F3 |
|  | E2F7 |
|  | E2F8 |
|  | ECM1 |
|  | ECT2 |
|  | EDIL3 |
|  | EDN1 |
|  | EDNRA |
|  | EFEMP2 |
|  | EFNA1 |
|  | EFNA4 |
|  | EFNA5 |
|  | EFNB2 |
|  | EGLN3 |
|  | EGR1 |
|  | EGR2 |
|  | EGR3 |
|  | EHF |
|  | EIF5A2 |
|  | EIF6 |
|  | ELF3 |
|  | ELK3 |
|  | ELOVL1 |
|  | ELOVL6 |
|  | EMB |
|  | EMP1 |
|  | EMP2 |
|  | EMP3 |
|  | ENC1 |
|  | ENDOD1 |
|  | ENO1 |
|  | ENO2 |
|  | ENOX1 |
|  | ENTPD1 |
|  | EPB41L1 |
|  | EPCAM |
|  | EPHA2 |
|  | EPHA4 |
|  | EPPK1 |
|  | EPS8 |
|  | EPS8L1 |
|  | EPS8L3 |
|  | EPSTI1 |
|  | EPYC |
|  | ERAP2 |
|  | ERBB2 |
|  | ERBB3 |
|  | EREG |
|  | ERN2 |
|  | ERO1A |
|  | ERV3-1 |
|  | ESM1 |
|  | ETHE1 |
|  | ETV1 |
|  | EXO1 |
|  | EXPH5 |
|  | EZH2 |
|  | EZR |
|  | F11R |
|  | F13A1 |
|  | F2R |
|  | F2RL1 |
|  | F2RL2 |
|  | F3 |
|  | F5 |
|  | FA2H |
|  | FAM135A |
|  | FAM156A |
|  | FAM177B |
|  | FAM3D |
|  | FAM72A |
|  | FAM72B |
|  | FAM72D |
|  | FAM83A |
|  | FAM83B |
|  | FAM83D |
|  | FAM83H |
|  | FANCD2 |
|  | FANCI |
|  | FANK1 |
|  | FAP |
|  | FAR2 |
|  | FAS |
|  | FAT1 |
|  | FBN1 |
|  | FBP1 |
|  | FBXL2 |
|  | FBXO32 |
|  | FCER1A |
|  | FCER1G |
|  | FCGR2A |
|  | FCGR2B |
|  | FCGR2C |
|  | FCGR3A |
|  | FCGR3B |
|  | FER1L4 |
|  | FER1L6 |
|  | FERMT1 |
|  | FGB |
|  | FGD6 |
|  | FGFBP1 |
|  | FHL2 |
|  | FHOD3 |
|  | FIBIN |
|  | FKBP10 |
|  | FKBP1A-SDCBP2 |
|  | FKBP7 |
|  | FLNA |
|  | FLNB |
|  | FLRT3 |
|  | FLVCR1 |
|  | FMN1 |
|  | FN1 |
|  | FNDC1 |
|  | FOXC1 |
|  | FOXD1 |
|  | FOXF2 |
|  | FOXM1 |
|  | FOXN2 |
|  | FOXQ1 |
|  | FRMD5 |
|  | FRMD6 |
|  | FRRS1 |
|  | FRZB |
|  | FSTL1 |
|  | FUNDC1 |
|  | FUT2 |
|  | FUT3 |
|  | FUT8 |
|  | FXYD3 |
|  | FXYD5 |
|  | FZD6 |
|  | FZD7 |
|  | GABRE |
|  | GABRP |
|  | GALE |
|  | GALNT3 |
|  | GALNT4 |
|  | GALNT5 |
|  | GAN |
|  | GAP43 |
|  | GAPDH |
|  | GAS5 |
|  | GASK1B |
|  | GATA3 |
|  | GBP1 |
|  | GBP1P1 |
|  | GBP2 |
|  | GBP3 |
|  | GBP4 |
|  | GBP5 |
|  | GCNT3 |
|  | GDA |
|  | GDI1 |
|  | GEM |
|  | GFPT2 |
|  | GJA1 |
|  | GJB2 |
|  | GJB3 |
|  | GJB6 |
|  | GLI2 |
|  | GLI3 |
|  | GLIPR1 |
|  | GLRX |
|  | GLT8D2 |
|  | GNA15 |
|  | GNB4 |
|  | GNB5 |
|  | GOLM1 |
|  | GPATCH2 |
|  | GPC6 |
|  | GPD2 |
|  | GPI |
|  | GPNMB |
|  | GPR137B |
|  | GPR35 |
|  | GPR87 |
|  | GPRC5A |
|  | GPSM2 |
|  | GPX2 |
|  | GPX8 |
|  | GREB1L |
|  | GREM1 |
|  | GRHL1 |
|  | GRN |
|  | GRP |
|  | GSDMB |
|  | GSDMC |
|  | GSDME |
|  | GSS |
|  | GSTP1 |
|  | GTF2IRD1 |
|  | GXYLT2 |
|  | H1-5 |
|  | H2AC13 |
|  | H2AC18 |
|  | H2AC19 |
|  | H2AC20 |
|  | H2AC21 |
|  | H2AC6 |
|  | H2BC12 |
|  | H2BC14 |
|  | H2BC18 |
|  | H2BC21 |
|  | H2BC5 |
|  | H2BP1 |
|  | H3C10 |
|  | H3C11 |
|  | H4C11 |
|  | H4C12 |
|  | H4C14 |
|  | H4C15 |
|  | HACD4 |
|  | HAS3 |
|  | HCAR3 |
|  | HCFC1R1 |
|  | HCP5 |
|  | HDAC1 |
|  | HELLS |
|  | HEPH |
|  | HERC6 |
|  | HES1 |
|  | HHIP |
|  | HIF1A |
|  | HILPDA |
|  | HK1 |
|  | HK2 |
|  | HKDC1 |
|  | HLA-A |
|  | HLA-B |
|  | HLA-C |
|  | HLA-DPA1 |
|  | HLA-DQA1 |
|  | HLA-DQA2 |
|  | HLA-DQB1 |
|  | HLA-DQB2 |
|  | HLA-DRA |
|  | HLA-DRB1 |
|  | HLA-DRB3 |
|  | HLA-DRB4 |
|  | HLA-DRB5 |
|  | HLA-E |
|  | HLA-F |
|  | HLA-G |
|  | HLA-H |
|  | HLA-J |
|  | HMCN1 |
|  | HMGA1 |
|  | HMGA2 |
|  | HMGB2 |
|  | HMMR |
|  | HOMER1 |
|  | HOPX |
|  | HOXA10 |
|  | HOXA13 |
|  | HOXB3 |
|  | HOXB5 |
|  | HOXB6 |
|  | HPGD |
|  | HRH1 |
|  | HS3ST1 |
|  | HS3ST3A1 |
|  | HSD17B2 |
|  | HSD17B6 |
|  | HSPA1A |
|  | HSPG2 |
|  | HSPH1 |
|  | HTATIP2 |
|  | HTRA1 |
|  | HTRA3 |
|  | IBSP |
|  | ICAM1 |
|  | ID1 |
|  | ID3 |
|  | IDO1 |
|  | IER3 |
|  | IER5L |
|  | IFI16 |
|  | IFI27 |
|  | IFI30 |
|  | IFI44 |
|  | IFI44L |
|  | IFI6 |
|  | IFIT1 |
|  | IFIT2 |
|  | IFIT3 |
|  | IFITM1 |
|  | IFITM10 |
|  | IFNE |
|  | IGF2BP2 |
|  | IGF2BP3 |
|  | IGFBP1 |
|  | IGFBP3 |
|  | IGFBP5 |
|  | IGFBP7 |
|  | IGFL2 |
|  | IGHD |
|  | IGHM |
|  | IGHV4-31 |
|  | IGKC |
|  | IGLJ3 |
|  | IGSF3 |
|  | IKZF2 |
|  | IL18 |
|  | IL1A |
|  | IL1R2 |
|  | IL1RAP |
|  | IL1RN |
|  | IL20RB |
|  | IL2RA |
|  | IL2RG |
|  | IL4R |
|  | IL7R |
|  | INAVA |
|  | INF2 |
|  | INHBA |
|  | INPP4B |
|  | INPP5F |
|  | INS |
|  | IQCE |
|  | IQGAP1 |
|  | IQGAP3 |
|  | IRAK2 |
|  | IRAK3 |
|  | IRF1 |
|  | IRF6 |
|  | IRS1 |
|  | ISG15 |
|  | ISL1 |
|  | ISLR |
|  | ISM1 |
|  | ITGA1 |
|  | ITGA11 |
|  | ITGA2 |
|  | ITGA3 |
|  | ITGA5 |
|  | ITGA6 |
|  | ITGAM |
|  | ITGAV |
|  | ITGB1 |
|  | ITGB2 |
|  | ITGB3 |
|  | ITGB4 |
|  | ITGB5 |
|  | ITGB6 |
|  | ITGB8 |
|  | ITGBL1 |
|  | ITPR3 |
|  | ITPRIPL2 |
|  | JAG1 |
|  | JMJD1C |
|  | JPT1 |
|  | JRKL |
|  | JUP |
|  | KCND2 |
|  | KCNE3 |
|  | KCNK1 |
|  | KCNK6 |
|  | KCNN4 |
|  | KCNS3 |
|  | KCTD10 |
|  | KDELR3 |
|  | KDM5B |
|  | KIAA1217 |
|  | KIAA1549L |
|  | KIF11 |
|  | KIF13B |
|  | KIF14 |
|  | KIF18A |
|  | KIF23 |
|  | KIF26B |
|  | KIF2C |
|  | KIF4A |
|  | KITLG |
|  | KLF5 |
|  | KLF6 |
|  | KLF7 |
|  | KLK10 |
|  | KLK6 |
|  | KLK7 |
|  | KNL1 |
|  | KNTC1 |
|  | KPNA2 |
|  | KRAS |
|  | KRT15 |
|  | KRT16 |
|  | KRT17 |
|  | KRT17P2 |
|  | KRT18 |
|  | KRT19 |
|  | KRT19P2 |
|  | KRT23 |
|  | KRT5 |
|  | KRT6A |
|  | KRT6B |
|  | KRT6C |
|  | KRT7 |
|  | KRT8 |
|  | KYNU |
|  | L3MBTL3 |
|  | LAD1 |
|  | LAMA3 |
|  | LAMA4 |
|  | LAMB1 |
|  | LAMB3 |
|  | LAMC2 |
|  | LAMP5 |
|  | LAPTM5 |
|  | LASP1 |
|  | LAYN |
|  | LBH |
|  | LCN2 |
|  | LCP1 |
|  | LDAF1 |
|  | LDHA |
|  | LEF1 |
|  | LEMD1 |
|  | LGALS1 |
|  | LGALS3 |
|  | LGALS3BP |
|  | LGALS4 |
|  | LGALS9 |
|  | LGALS9C |
|  | LGR5 |
|  | LIF |
|  | LIMA1 |
|  | LIMS1 |
|  | LIMS3 |
|  | LIMS3-LOC440895 |
|  | LIMS4 |
|  | LINC01559 |
|  | LIPH |
|  | LMBR1 |
|  | LMNA |
|  | LMO4 |
|  | LMO7 |
|  | LNPK |
|  | LOX |
|  | LOXL1 |
|  | LOXL2 |
|  | LPAR5 |
|  | LPCAT2 |
|  | LPCAT4 |
|  | LPP |
|  | LRCH1 |
|  | LRIG3 |
|  | LRP1 |
|  | LRP12 |
|  | LRP8 |
|  | LRRC1 |
|  | LRRC15 |
|  | LRRC17 |
|  | LRRC8A |
|  | LRRFIP1 |
|  | LRRN1 |
|  | LSR |
|  | LTBP1 |
|  | LTBP2 |
|  | LUM |
|  | LXN |
|  | LY6D |
|  | LY6E |
|  | LY75 |
|  | LY75-CD302 |
|  | LYPD1 |
|  | LYZ |
|  | LZTS1 |
|  | MACC1 |
|  | MAD2L1 |
|  | MAGI2 |
|  | MAL2 |
|  | MALL |
|  | MAP4K4 |
|  | MAP9 |
|  | MARCKS |
|  | MARVELD1 |
|  | MATN3 |
|  | MBOAT1 |
|  | MBOAT2 |
|  | MCM4 |
|  | MCM6 |
|  | MCU |
|  | MCUB |
|  | MDK |
|  | MEAK7 |
|  | MEGF6 |
|  | MELK |
|  | MELTF |
|  | MET |
|  | MFAP2 |
|  | MFAP5 |
|  | MFGE8 |
|  | MFSD6 |
|  | MGAT4B |
|  | MGLL |
|  | MGP |
|  | MIA |
|  | MIA-RAB4B |
|  | MICAL2 |
|  | MICALL2 |
|  | MICOS10-NBL1 |
|  | MIF |
|  | MINDY2 |
|  | MIR31HG |
|  | MIR4435-2HG |
|  | MISP |
|  | MKI67 |
|  | MLF1 |
|  | MLPH |
|  | MME |
|  | MMP1 |
|  | MMP10 |
|  | MMP11 |
|  | MMP12 |
|  | MMP13 |
|  | MMP14 |
|  | MMP2 |
|  | MMP28 |
|  | MMP3 |
|  | MMP7 |
|  | MMP9 |
|  | MNS1 |
|  | MOB1A |
|  | MOCOS |
|  | MOXD1 |
|  | MPZL2 |
|  | MR1 |
|  | MRC1 |
|  | MROH6 |
|  | MRPS6 |
|  | MSANTD3-TMEFF1 |
|  | MSL3P1 |
|  | MSLN |
|  | MSMB |
|  | MSMO1 |
|  | MSN |
|  | MSR1 |
|  | MSRB3 |
|  | MST1R |
|  | MSX2 |
|  | MTMR11 |
|  | MUC1 |
|  | MUC13 |
|  | MUC16 |
|  | MUC17 |
|  | MUC20 |
|  | MUC3A |
|  | MUC3B |
|  | MUC4 |
|  | MUC5AC |
|  | MUC5B |
|  | MUCL3 |
|  | MVP |
|  | MX1 |
|  | MX2 |
|  | MXD1 |
|  | MXRA5 |
|  | MXRA8 |
|  | MYB |
|  | MYBPC1 |
|  | MYEOV |
|  | MYH9 |
|  | MYL12A |
|  | MYL12B |
|  | MYLK |
|  | MYO1D |
|  | MYO1E |
|  | MYO6 |
|  | MYOF |
|  | NALCN |
|  | NAMPT |
|  | NAPG |
|  | NBL1 |
|  | NCAPG |
|  | NCAPG2 |
|  | NCEH1 |
|  | NCF2 |
|  | NCK1 |
|  | NDC80 |
|  | NDE1 |
|  | NDNF |
|  | NDP |
|  | NDRG1 |
|  | NEB |
|  | NECTIN4 |
|  | NEK2 |
|  | NEK7 |
|  | NET1 |
|  | NFE2L1 |
|  | NFE2L3 |
|  | NID1 |
|  | NME7 |
|  | NMI |
|  | NMU |
|  | NMUR2 |
|  | NNMT |
|  | NOX4 |
|  | NPAS2 |
|  | NPC1 |
|  | NPR3 |
|  | NPSR1 |
|  | NQO1 |
|  | NREP |
|  | NRP2 |
|  | NT5DC2 |
|  | NT5E |
|  | NTM |
|  | NUAK1 |
|  | NUF2 |
|  | NUP107 |
|  | OAS1 |
|  | OAS2 |
|  | OAS3 |
|  | OASL |
|  | OCIAD2 |
|  | ODAM |
|  | OLFM4 |
|  | OLFML2B |
|  | OLR1 |
|  | ONECUT2 |
|  | OPN3 |
|  | OSBPL10 |
|  | OSBPL3 |
|  | OSMR |
|  | OSR2 |
|  | OXCT1 |
|  | OXR1 |
|  | OXTR |
|  | P3H2 |
|  | P4HA1 |
|  | PABPC1 |
|  | PABPC1L |
|  | PADI1 |
|  | PAK1 |
|  | PALLD |
|  | PARD6B |
|  | PARP12 |
|  | PARP14 |
|  | PARP4 |
|  | PARP9 |
|  | PAWR |
|  | PAX8-AS1 |
|  | PBK |
|  | PCDH7 |
|  | PCDHB10 |
|  | PCDHB14 |
|  | PCDHB16 |
|  | PCDHB9 |
|  | PCLAF |
|  | PCSK5 |
|  | PDCD1LG2 |
|  | PDCD6 |
|  | PDGFC |
|  | PDGFRB |
|  | PDGFRL |
|  | PDLIM3 |
|  | PDLIM4 |
|  | PDLIM7 |
|  | PDPN |
|  | PDZK1IP1 |
|  | PELI1 |
|  | PELO |
|  | PERP |
|  | PFKP |
|  | PGAM1 |
|  | PGK1 |
|  | PGM2 |
|  | PGM2L1 |
|  | PHACTR3 |
|  | PHLDA1 |
|  | PHLDA2 |
|  | PHLDA3 |
|  | PI3 |
|  | PIAS3 |
|  | PIGB |
|  | PIGR |
|  | PIK3IP1 |
|  | PJA1 |
|  | PKIB |
|  | PKM |
|  | PKP4 |
|  | PLA2G10 |
|  | PLA2G4A |
|  | PLA2G7 |
|  | PLA2R1 |
|  | PLAAT3 |
|  | PLAAT4 |
|  | PLAC8 |
|  | PLAT |
|  | PLAU |
|  | PLAUR |
|  | PLBD1 |
|  | PLCB4 |
|  | PLEC |
|  | PLEK2 |
|  | PLEKHA2 |
|  | PLEKHA6 |
|  | PLEKHA7 |
|  | PLEKHB2 |
|  | PLEKHG1 |
|  | PLIN3 |
|  | PLK1 |
|  | PLK2 |
|  | PLOD1 |
|  | PLOD2 |
|  | PLOD3 |
|  | PLPP4 |
|  | PLS1 |
|  | PLS3 |
|  | PLXDC1 |
|  | PLXDC2 |
|  | PLXNA1 |
|  | PLXNC1 |
|  | PMAIP1 |
|  | PMEPA1 |
|  | PMP22 |
|  | PNMA1 |
|  | PNP |
|  | POC1B-GALNT4 |
|  | PODXL |
|  | POF1B |
|  | POGLUT2 |
|  | PON2 |
|  | POSTN |
|  | PPARG |
|  | PPEF1 |
|  | PPFIA2 |
|  | PPFIBP1 |
|  | PPIC |
|  | PPL |
|  | PPP1R13L |
|  | PPP1R14C |
|  | PPP1R3B |
|  | PPP2R2C |
|  | PRC1 |
|  | PRDM1 |
|  | PRDX1 |
|  | PRELP |
|  | PRICKLE1 |
|  | PRKACB |
|  | PRKCI |
|  | PROM1 |
|  | PROM2 |
|  | PRR11 |
|  | PRR15 |
|  | PRR5L |
|  | PRRG1 |
|  | PRRG4 |
|  | PRRX1 |
|  | PRSS23 |
|  | PRTFDC1 |
|  | PSCA |
|  | PSD3 |
|  | PSEN1 |
|  | PSMB8 |
|  | PSMB9 |
|  | PTBP3 |
|  | PTGES |
|  | PTGIS |
|  | PTGS2 |
|  | PTK6 |
|  | PTK7 |
|  | PTPN12 |
|  | PTPN13 |
|  | PTPN14 |
|  | PTPRA |
|  | PTPRE |
|  | PTPRF |
|  | PTPRH |
|  | PTPRK |
|  | PTPRR |
|  | PTTG1 |
|  | PTTG1IP |
|  | PXDN |
|  | PYGB |
|  | PYGL |
|  | QPCT |
|  | QSOX1 |
|  | RAB11A |
|  | RAB23 |
|  | RAB25 |
|  | RAB27B |
|  | RAB31 |
|  | RAB34 |
|  | RAB3B |
|  | RAC1 |
|  | RACGAP1 |
|  | RAD51AP1 |
|  | RAI1 |
|  | RAI14 |
|  | RALA |
|  | RALB |
|  | RAPGEFL1 |
|  | RAPH1 |
|  | RARRES1 |
|  | RASA1 |
|  | RASA3 |
|  | RASAL2 |
|  | RASEF |
|  | RASGEF1A |
|  | RASGRF2 |
|  | RASSF6 |
|  | RBBP8 |
|  | RBM12B |
|  | RBMS1 |
|  | RCC2 |
|  | RCN1 |
|  | RCN3 |
|  | RECQL |
|  | REEP3 |
|  | REG4 |
|  | RFTN1 |
|  | RFX5 |
|  | RGS1 |
|  | RGS16 |
|  | RGS17 |
|  | RGS4 |
|  | RHBDF1 |
|  | RHBDL2 |
|  | RHOC |
|  | RHOQ |
|  | RHPN2 |
|  | RIOK3 |
|  | RIT1 |
|  | RNASET2 |
|  | RND3 |
|  | RNF128 |
|  | RNF149 |
|  | RNF183 |
|  | RNF19B |
|  | RNF213 |
|  | RPGRIP1L |
|  | RRAS |
|  | RRM2 |
|  | RSAD2 |
|  | RTKN2 |
|  | RTP4 |
|  | RUNX1 |
|  | RUNX2 |
|  | RUVBL1 |
|  | S100A10 |
|  | S100A11 |
|  | S100A14 |
|  | S100A16 |
|  | S100A2 |
|  | S100A4 |
|  | S100A6 |
|  | S100P |
|  | SALL4 |
|  | SAMD12 |
|  | SAMD9 |
|  | SAMD9L |
|  | SCEL |
|  | SCGB2A1 |
|  | SCIN |
|  | SCNN1A |
|  | SCRN1 |
|  | SCUBE3 |
|  | SDC1 |
|  | SDC4 |
|  | SDCBP2 |
|  | SDR16C5 |
|  | SEC14L2 |
|  | SEC23A |
|  | SEMA3A |
|  | SEMA3C |
|  | SEMA4B |
|  | SEMA7A |
|  | SEPTIN9 |
|  | SERINC2 |
|  | SERPINA1 |
|  | SERPINB2 |
|  | SERPINB3 |
|  | SERPINB4 |
|  | SERPINB5 |
|  | SERPINB9 |
|  | SERPINE1 |
|  | SERPINH1 |
|  | SERTAD4 |
|  | SESTD1 |
|  | SFN |
|  | SFRP2 |
|  | SFRP4 |
|  | SFTA2 |
|  | SGIP1 |
|  | SGMS2 |
|  | SGPP2 |
|  | SH3KBP1 |
|  | SH3PXD2A |
|  | SH3PXD2B |
|  | SH3RF1 |
|  | SHCBP1 |
|  | SHOX2 |
|  | SHROOM3 |
|  | SIX1 |
|  | SKAP2 |
|  | SKIL |
|  | SLC10A3 |
|  | SLC11A1 |
|  | SLC12A2 |
|  | SLC12A8 |
|  | SLC16A1 |
|  | SLC16A3 |
|  | SLC16A4 |
|  | SLC16A5 |
|  | SLC16A6 |
|  | SLC1A1 |
|  | SLC20A1 |
|  | SLC22A3 |
|  | SLC24A3 |
|  | SLC25A12 |
|  | SLC25A13 |
|  | SLC25A24 |
|  | SLC26A9 |
|  | SLC28A3 |
|  | SLC2A1 |
|  | SLC2A3 |
|  | SLC35F2 |
|  | SLC39A10 |
|  | SLC44A1 |
|  | SLC44A4 |
|  | SLC45A3 |
|  | SLC4A11 |
|  | SLC5A3 |
|  | SLC6A14 |
|  | SLC6A20 |
|  | SLC6A6 |
|  | SLC7A11 |
|  | SLC7A7 |
|  | SLC9A2 |
|  | SLC9B2 |
|  | SLCO1B3 |
|  | SLCO1B7 |
|  | SLCO4A1 |
|  | SLFN13 |
|  | SLFN5 |
|  | SLITRK6 |
|  | SLK |
|  | SLPI |
|  | SMAD3 |
|  | SMAGP |
|  | SMC4 |
|  | SMC6 |
|  | SMOC2 |
|  | SMS |
|  | SMURF1 |
|  | SNAI2 |
|  | SNHG12 |
|  | SNX7 |
|  | SOCS6 |
|  | SOX11 |
|  | SOX4 |
|  | SOX9 |
|  | SP140L |
|  | SPAG1 |
|  | SPARC |
|  | SPATS2L |
|  | SPDL1 |
|  | SPHK1 |
|  | SPIN4 |
|  | SPINK5 |
|  | SPINT1 |
|  | SPINT2 |
|  | SPOCK1 |
|  | SPON1 |
|  | SPON2 |
|  | SPP1 |
|  | SPRED1 |
|  | SPRR1B |
|  | SPRR3 |
|  | SPRY4 |
|  | SPTLC2 |
|  | SQLE |
|  | SQOR |
|  | SRD5A3 |
|  | SRGAP1 |
|  | SRGAP2 |
|  | SRGAP2B |
|  | SRGAP2C |
|  | SRPX2 |
|  | SSPN |
|  | ST14 |
|  | ST6GALNAC1 |
|  | STAMBPL1 |
|  | STAT1 |
|  | STAT2 |
|  | STEAP1 |
|  | STEAP2 |
|  | STIL |
|  | STING1 |
|  | STK3 |
|  | STK38 |
|  | STK38L |
|  | STMN1 |
|  | STMN2 |
|  | STRA6 |
|  | STX1A |
|  | STX6 |
|  | STYK1 |
|  | SUGCT |
|  | SULF1 |
|  | SULF2 |
|  | SULT1B1 |
|  | SULT1C2 |
|  | SUSD2 |
|  | SYT13 |
|  | SYT17 |
|  | SYTL2 |
|  | SYTL4 |
|  | TACSTD2 |
|  | TAF1D |
|  | TAGLN |
|  | TAGLN2 |
|  | TANC2 |
|  | TAP1 |
|  | TAP2 |
|  | TBC1D2 |
|  | TCF3 |
|  | TCF7L2 |
|  | TCN1 |
|  | TDO2 |
|  | TES |
|  | TFAP2A |
|  | TFF1 |
|  | TFF2 |
|  | TFF3 |
|  | TFPI |
|  | TFRC |
|  | TGFA |
|  | TGFB1 |
|  | TGFB2 |
|  | TGFBI |
|  | TGFBR1 |
|  | TGIF1 |
|  | TGM2 |
|  | THBS1 |
|  | THBS2 |
|  | THBS4 |
|  | THY1 |
|  | TIAM2 |
|  | TIMP1 |
|  | TIMP2 |
|  | TIMP3 |
|  | TINAGL1 |
|  | TJP2 |
|  | TM4SF1 |
|  | TM4SF4 |
|  | TMC5 |
|  | TMC7 |
|  | TMEM128 |
|  | TMEM154 |
|  | TMEM165 |
|  | TMEM176A |
|  | TMEM184B |
|  | TMEM200A |
|  | TMEM44 |
|  | TMEM45A |
|  | TMEM45B |
|  | TMEM54 |
|  | TMEM87A |
|  | TMEM87B |
|  | TMOD3 |
|  | TMPRSS3 |
|  | TMPRSS4 |
|  | TMSB10 |
|  | TMSB15B |
|  | TMTC2 |
|  | TNC |
|  | TNFAIP2 |
|  | TNFAIP6 |
|  | TNFRSF10A |
|  | TNFRSF10B |
|  | TNFRSF11A |
|  | TNFRSF11B |
|  | TNFRSF21 |
|  | TNFSF11 |
|  | TNFSF4 |
|  | TNIK |
|  | TNS4 |
|  | TOP2A |
|  | TOX3 |
|  | TP53I3 |
|  | TPBG |
|  | TPD52L2 |
|  | TPI1 |
|  | TPM1 |
|  | TPM2 |
|  | TPM4 |
|  | TPRG1 |
|  | TPX2 |
|  | TRAK1 |
|  | TRANK1 |
|  | TREM1 |
|  | TREM2 |
|  | TRERF1 |
|  | TRIB2 |
|  | TRIM16 |
|  | TRIM16L |
|  | TRIM22 |
|  | TRIM29 |
|  | TRIM31 |
|  | TRIM56 |
|  | TRIM59 |
|  | TRIM6 |
|  | TRIO |
|  | TRIP10 |
|  | TRIP6 |
|  | TRPC1 |
|  | TRPS1 |
|  | TSHZ3 |
|  | TSPAN1 |
|  | TSPAN15 |
|  | TSPAN2 |
|  | TSPAN5 |
|  | TSPAN8 |
|  | TTC39A |
|  | TTC8 |
|  | TTK |
|  | TTLL12 |
|  | TUBA1C |
|  | TUBA4A |
|  | TUBB |
|  | TUBB3 |
|  | TUFT1 |
|  | TWSG1 |
|  | TXN |
|  | TYMS |
|  | UBA6 |
|  | UBASH3B |
|  | UBD |
|  | UBE2C |
|  | UBE2T |
|  | UCA1 |
|  | UCHL1 |
|  | UGT1A1 |
|  | UGT1A10 |
|  | UGT1A3 |
|  | UGT1A4 |
|  | UGT1A5 |
|  | UGT1A6 |
|  | UGT1A7 |
|  | UGT1A8 |
|  | UGT1A9 |
|  | UGT8 |
|  | UHMK1 |
|  | UHRF1 |
|  | UNC5B |
|  | UNC5CL |
|  | USH1C |
|  | USP18 |
|  | USP54 |
|  | VASP |
|  | VCAN |
|  | VCL |
|  | VDR |
|  | VEGFA |
|  | VGLL1 |
|  | VILL |
|  | VNN1 |
|  | VPS37B |
|  | VPS41 |
|  | VRK1 |
|  | VSIG1 |
|  | VSIG2 |
|  | VSNL1 |
|  | WDFY1 |
|  | WDHD1 |
|  | WDR1 |
|  | WFDC2 |
|  | WIPF1 |
|  | WNK1 |
|  | WNT2 |
|  | WNT5A |
|  | XAF1 |
|  | XDH |
|  | XRCC4 |
|  | XYLT1 |
|  | YEATS2 |
|  | YWHAH |
|  | YWHAZ |
|  | ZBTB7A |
|  | ZC2HC1A |
|  | ZC3H12A |
|  | ZDHHC13 |
|  | ZDHHC20 |
|  | ZDHHC3 |
|  | ZDHHC7 |
|  | ZEB1 |
|  | ZFC3H1 |
|  | ZFP36L2 |
|  | ZG16B |
|  | ZMYM2 |
|  | ZMYND8 |
|  | ZNF117 |
|  | ZNF185 |
|  | ZNF217 |
|  | ZNF292 |
|  | ZNF365 |
|  | ZNF521 |
|  | ZNF532 |
|  | ZNF83 |
|  | ZNF860 |
|  | ZNFX1 |
|  | ZWILCH |
|  | ZWINT |
