# Supplementary material for: Leucine rich repeat LGI family member 3: Integrative analyses support its prognostic association with pancreatic adenocarcinoma
Source: Medicine (Baltimore). 2024 Feb 23;103(8):e37183. doi: 10.1097/MD.0000000000037183 (PMC11309673; doi:10.1097/MD.0000000000037183)
Supplement: Supplementary file 3 [file medi-103-e37183-s003.docx]

Table S3. List of LGI3-regulated genes reported in the previous literatures ^[1-18]^.

| Groups | Gene name |
| --- | --- |
| Upregulated by LGI3 | ADCK2 |
|  | ADGRE1 |
|  | AFP |
|  | AKT1 |
|  | BLNK |
|  | BRCA1 |
|  | BTK |
|  | CALM1 |
|  | CASP1 |
|  | CCL11 |
|  | CCL12 |
|  | CCL2 |
|  | CCL21 |
|  | CD37 |
|  | CD63 |
|  | CD68 |
|  | CD80 |
|  | CREB1 |
|  | CSF3 |
|  | CTNNB1 |
|  | CXCL13 |
|  | CXCL2 |
|  | CXCL5 |
|  | CYBA |
|  | CYBB |
|  | E2F2 |
|  | ERBB2 |
|  | ERBB3 |
|  | ERN1 |
|  | F10 |
|  | F12 |
|  | F3 |
|  | FLG |
|  | FOXO3 |
|  | GAD1 |
|  | GAS6 |
|  | GH1 |
|  | GPR151 |
|  | GRK2 |
|  | IGF1 |
|  | IGFBP5 |
|  | IKBKB |
|  | IL2RA |
|  | IL6 |
|  | ITGAX |
|  | IVL |
|  | JUN |
|  | KRT10 |
|  | LIMK1 |
|  | LOR |
|  | LYN |
|  | MAPK1 |
|  | MAPK3 |
|  | MDM2 |
|  | MITF |
|  | MLH1 |
|  | MSTN |
|  | NCF1 |
|  | NCF2 |
|  | NFKB1 |
|  | NOS2 |
|  | PIK3CA |
|  | PRKAA1 |
|  | PTEN |
|  | PTGS1 |
|  | PTGS2 |
|  | PTK2 |
|  | PTK6 |
|  | RCBTB1 |
|  | RELA |
|  | RPS27 |
|  | RPS6KA1 |
|  | SEMA4D |
|  | SLC3A2 |
|  | SND1 |
|  | TGM1 |
|  | TIMP1 |
|  | TNF |
|  | TNFSF13B |
|  | TP73 |
|  | TUBB3 |
|  | TYK2 |
|  | TYRO3 |
| Downregulated by LGI3 | AHSG |
|  | ADCK1 |
|  | ADIPOQ |
|  | AKT2 |
|  | ALK |
|  | AXL |
|  | BAD |
|  | C1S |
|  | C5 |
|  | CAMKV |
|  | CAV1 |
|  | CBL |
|  | CCL6 |
|  | CD247 |
|  | CD3E |
|  | CD5 |
|  | CEBPA |
|  | CFB |
|  | CFD |
|  | COL4A3 |
|  | CRP |
|  | CRYAB |
|  | CSF1 |
|  | CST3 |
|  | CYP2S1 |
|  | CYP39A1 |
|  | DCC |
|  | DDX4 |
|  | DLK1 |
|  | DNAL4 |
|  | DOK1 |
|  | DPP4 |
|  | EEF1G |
|  | EGF |
|  | EIF4EBP1 |
|  | EPHB1 |
|  | EPN3 |
|  | ESM1 |
|  | ESR1 |
|  | EXOG |
|  | F2R |
|  | FABP4 |
|  | FGF1 |
|  | FLI1 |
|  | FN1 |
|  | FOXA2 |
|  | GSK3A |
|  | GSK3B |
|  | HSP90AB1 |
|  | IGFBP1 |
|  | IGFBP2 |
|  | IGFBP6 |
|  | INSR |
|  | IRS1 |
|  | KAT8 |
|  | KDR |
|  | KIT |
|  | KLK3 |
|  | KRT18 |
|  | LAMC3 |
|  | LPL |
|  | MAP2K2 |
|  | MAPK14 |
|  | MATK |
|  | MTOR |
|  | MUC16 |
|  | NCR1 |
|  | NEUROG3 |
|  | NFKBIA |
|  | PDGFRA |
|  | POLR3D |
|  | POSTN |
|  | POU3F1 |
|  | PPARG |
|  | PRKCA |
|  | PRKCD |
|  | PRL2C2 |
|  | PRPF19 |
|  | RARRES2 |
|  | RCHY1 |
|  | REG3G |
|  | RETN |
|  | RYR2 |
|  | SERPINE1 |
|  | SLU7 |
|  | SNAI2 |
|  | STMN1 |
|  | STX1A |
|  | TAF4 |
|  | TBP |
|  | TP53 |
|  | TP63 |
|  | USP13 |
|  | ZAP70 |
