# Supplementary material for: Leucine rich repeat LGI family member 3: Integrative analyses support its prognostic association with pancreatic adenocarcinoma
Source: Medicine (Baltimore). 2024 Feb 23;103(8):e37183. doi: 10.1097/MD.0000000000037183 (PMC11309673; doi:10.1097/MD.0000000000037183)
Supplement: Supplementary file 4 [file medi-103-e37183-s004.docx]

Table S4. List of genes in Figure 2.

|  |  |  |
| --- | --- | --- |
| Groups |  | Gene name |
| + |  | CCL2 |
|  |  | CXCL2 |
|  |  | F10 |
|  |  | IGF1 |
|  |  | IL6 |
|  |  | NOS2 |
| x |  | AXL |
|  |  | C1S |
|  |  | CYP2S1 |
|  |  | ESM1 |
|  |  | F2R |
|  |  | FN1 |
|  |  | IGFBP1 |
|  |  | IRS1 |
|  |  | KRT18 |
|  |  | MUC16 |
|  |  | POSTN |
|  |  | PPARG |
|  |  | SERPINE1 |
|  |  | SNAI2 |
|  |  | STMN1 |
|  |  | STX1A |
| * |  | CD68 |
|  |  | CD80 |
|  |  | CTNNB1 |
|  |  | CXCL5 |
|  |  | CYBB |
|  |  | ERBB2 |
|  |  | ERBB3 |
|  |  | F3 |
|  |  | IGFBP5 |
|  |  | IL2RA |
|  |  | NCF2 |
|  |  | PTGS2 |
|  |  | PTK6 |
|  |  | TIMP1 |
|  |  | TUBB3 |
| # |  | ADIPOQ |
|  |  | C5 |
|  |  | CFD |
|  |  | CRYAB |
|  |  | CYP39A1 |
|  |  | DLK1 |
|  |  | EGF |
|  |  | FABP4 |
|  |  | IGFBP2 |
|  |  | KLK3 |
|  |  | RARRES2 |
|  |  | REG3G |
|  |  | RYR2 |
