# Supplementary material for: Leucine rich repeat LGI family member 3: Integrative analyses support its prognostic association with pancreatic adenocarcinoma
Source: Medicine (Baltimore). 2024 Feb 23;103(8):e37183. doi: 10.1097/MD.0000000000037183 (PMC11309673; doi:10.1097/MD.0000000000037183)
Supplement: Supplementary file 5 [file medi-103-e37183-s005.docx]

Table S5. The transcription factor affinity prediction table of the genes in groups b, c and d in Figure 3A. (P<0.01)

|  | |  |  |  |
| --- | --- | --- | --- | --- |
| Matrix | Transcription Factor | | | Association Score |
| CHCH_01 | Chch | | | 21.838 |
| TFIII_Q6 | Tfii-i | | | 16.537 |
| E2F_Q2 | Dp-1, E2f-1 | | | 15.061 |
| SP1_Q2_01 | Sp1, Sp2 | | | 14.615 |
| MAZ_Q6 | Maz | | | 13.309 |
| AP2_Q6 | Ap-2, Ap-2alpha | | | 12.279 |
| MOVOB_01 | Movo-b | | | 12.279 |
| LRF_Q2 | Fbi-1, Lrf | | | 10.976 |
| ZF5_01 | Zf5 | | | 10.122 |
| MZF1_01 | Mzf-1 | | | 9.492 |
| EGR_Q6 | Egr-1, Egr-2 | | | 9.334 |
| AP2GAMMA_01 | Ap-2gamma | | | 8.941 |
| DEAF1_01 | Deaf-1 | | | 7.985 |
| MAZR_01 | Mazr | | | 7.603 |
| CREB_Q2_01 | Creb, Crebbeta | | | 6.9 |
| SPZ1_01 | Spz1 | | | 6.624 |
| CREBP1CJUN_01 | Atf-2, C-jun | | | 6.493 |
| CREB_01 | Creb, Deltacreb | | | 6.493 |
| ATF3_Q6 | Atf3 | | | 6.168 |
| MYCMAX_B | Max2, C-myc | | | 6.157 |
| SREBP1_Q6 | Srebp-1, Srebp-1c | | | 5.892 |
| HIC1_02 | Hic-1 | | | 5.739 |
| NFKB_Q6_01 | Nf-kappab1, Nf-kappab2 | | | 5.472 |
| CREBP1_Q2 | Atf-2, Cre-bp1 | | | 5.424 |
| ATF4_Q2 | Atf-4, Atf4 | | | 5.358 |
| ZIC3_01 | Zic3 | | | 5.223 |
| CREB_Q3 | Atf-1, Atf-2 | | | 5.052 |
| PAX5_01 | Pax-5 | | | 4.846 |
| HES1_Q2 | Hes-1 | | | 4.541 |
| NERF_Q2 | Nerf-1a | | | 4.52 |
| TAL1ALPHAE47_01 | E47, Tal-1alpha | | | 4.503 |
| MTF1_Q4 | Mtf-1 | | | 4.244 |
| USF2_Q6 | Usf2a | | | 4.244 |
| VDR_Q3 | Vdr | | | 4.234 |
| PAX4_01 | Pax-4a | | | 4.136 |
| EGR3_01 | Egr-3 | | | 4.009 |
| NGFIC_01 | Egr-4 | | | 4.009 |
| AP1_01 | Fosb, Fra-1 | | | 3.9 |
| NF1_Q6 | Nf-1 | | | 3.89 |
| RREB1_01 | Rreb-1 | | | 3.876 |
| TAL1_Q6 | Tal-1, Tal-1alpha | | | 3.771 |
