# Supplementary material for: Leucine rich repeat LGI family member 3: Integrative analyses support its prognostic association with pancreatic adenocarcinoma
Source: Medicine (Baltimore). 2024 Feb 23;103(8):e37183. doi: 10.1097/MD.0000000000037183 (PMC11309673; doi:10.1097/MD.0000000000037183)
Supplement: Supplementary file 6 [file medi-103-e37183-s006.docx]

Table S6. List of genes in Figure 4A and B.

|  |  |
| --- | --- |
| Groups | Gene name |
| + | BLNK |
|  | CALM1 |
|  | CASP1 |
|  | CCL11 |
|  | CCL2 |
|  | CXCL13 |
|  | F3 |
|  | GAS6 |
|  | IGFBP5 |
|  | PTGS1 |
|  | TNFSF13B |
| x | AHSG |
|  | C5 |
|  | CBL |
|  | FABP4 |
|  | IGFBP1 |
|  | MUC16 |
|  | PPARG |
| * | F12 |
|  | PTK6 |
| # | NEUROG3 |
|  | C1S |
|  | KIT |
|  | EGF |
|  | POSTN |
|  | RARRES2 |
|  | REG3G |
