# Supplementary material for: Leucine rich repeat LGI family member 3: Integrative analyses support its prognostic association with pancreatic adenocarcinoma
Source: Medicine (Baltimore). 2024 Feb 23;103(8):e37183. doi: 10.1097/MD.0000000000037183 (PMC11309673; doi:10.1097/MD.0000000000037183)
Supplement: Supplementary file 7 [file medi-103-e37183-s007.docx]

Table S7. List of genes in Figure 7.

|  |  |
| --- | --- |
| Groups | Gene name |
| LGI3-upregulated | ADCK1 |
| Favorable | ADIPOQ |
|  | ALK |
|  | C5 |
|  | CAMKV |
|  | CD247 |
|  | CFD |
|  | CST3 |
|  | DCC |
|  | DOK1 |
|  | EXOG |
|  | FLI1 |
|  | FOXA2 |
|  | GSK3A |
|  | IGFBP2 |
|  | KDR |
|  | LPL |
|  | MAP2K2 |
|  | MATK |
|  | NEUROG3 |
|  | POU3F1 |
|  | RCHY1 |
|  | TBP |
|  | USP13 |
|  | ZAP70 |
| LGI3-upregulated | AXL |
| Unfavorable | CAV1 |
|  | CBL |
|  | CFB |
|  | CRP |
|  | CYP2S1 |
|  | EGF |
|  | EIF4EBP1 |
|  | EPN3 |
|  | FN1 |
|  | GSK3B |
|  | IGFBP6 |
|  | IRS1 |
|  | KLK3 |
|  | KRT18 |
|  | MTOR |
|  | MUC16 |
|  | POSTN |
|  | PPARG |
|  | RARRES2 |
|  | SERPINE1 |
|  | SNAI2 |
|  | TP63 |
| LGI3-downregulated | ADCK2 |
| Favorable | ADGRE1 |
|  | AKT1 |
|  | CALM1 |
|  | CCL21 |
|  | CD63 |
|  | F10 |
|  | GH1 |
|  | KRT10 |
|  | MSTN |
|  | NCF1 |
|  | RCBTB1 |
|  | SEMA4D |
|  | SLC3A2 |
|  | TNF |
|  | TUBB3 |
|  | TYK2 |
| LGI3-downregulated | BRCA1 |
| Unfavorable | CASP1 |
|  | CCL11 |
|  | CD80 |
|  | CTNNB1 |
|  | CXCL5 |
|  | CYBA |
|  | E2F2 |
|  | ERBB2 |
|  | ERBB3 |
|  | F3 |
|  | FOXO3 |
|  | IL6 |
|  | IVL |
|  | LIMK1 |
|  | LYN |
|  | MAPK1 |
|  | MDM2 |
|  | MITF |
|  | NOS2 |
|  | PIK3CA |
|  | PRKAA1 |
|  | PTGS2 |
|  | PTK2 |
|  | PTK6 |
|  | RPS27 |
|  | RPS6KA1 |
|  | TNFSF13B |
