# Supplementary material for: Leucine rich repeat LGI family member 3: Integrative analyses support its prognostic association with pancreatic adenocarcinoma
Source: Medicine (Baltimore). 2024 Feb 23;103(8):e37183. doi: 10.1097/MD.0000000000037183 (PMC11309673; doi:10.1097/MD.0000000000037183)
Supplement: Supplementary file 8 [file medi-103-e37183-s008.docx]

Table S8. Functional enrichment analysis of the groups of gene products in Figure 7. (P<0.01 or top 20 lowest P-value)

| Category | Term | Count | P-value |
| --- | --- | --- | --- |
| LGI3-upregulated, favorable | | | |
| GOTERM_MF_DIRECT | GO:0004713~protein tyrosine kinase activity | 5 | 1.18x10^-5^ |
| GOTERM_MF_DIRECT | GO:0004714~transmembrane receptor protein tyrosine kinase activity | 5 | 1.31x10^-5^ |
| GOTERM_MF_DIRECT | GO:0005102~receptor binding | 6 | 1.23x10^-4^ |
| GOTERM_BP_DIRECT | GO:0007169~transmembrane receptor protein tyrosine kinase signaling pathway | 4 | 5.82x10^-4^ |
| GOTERM_BP_DIRECT | GO:0018108~peptidyl-tyrosine phosphorylation | 4 | 7.18x10^-4^ |
| KEGG_PATHWAY | hsa05235:PD-L1 expression and PD-1 checkpoint pathway in cancer | 4 | 7.63x10^-4^ |
| GOTERM_BP_DIRECT | GO:0045893~positive regulation of transcription, DNA-templated | 6 | 1.73x10^-3^ |
| GOTERM_CC_DIRECT | GO:0005576~extracellular region | 9 | 2.09x10^-3^ |
| GOTERM_MF_DIRECT | GO:0005524~ATP binding | 8 | 2.43x10^-3^ |
| GOTERM_BP_DIRECT | GO:0000187~activation of MAPK activity | 3 | 3.69x10^-3^ |
| GOTERM_BP_DIRECT | GO:2000467~positive regulation of glycogen (starch) synthase activity | 2 | 6.20x10^-3^ |
| GOTERM_BP_DIRECT | GO:0009617~response to bacterium | 3 | 9.82x10^-3^ |
| GOTERM_BP_DIRECT | GO:2000171~negative regulation of dendrite development | 2 | 9.90x10^-3^ |
| LGI3-upregulated, unfavorable | |  |  |
| GOTERM_CC_DIRECT | GO:0005615~extracellular space | 11 | 1.21x10^-5^ |
| KEGG_PATHWAY | hsa01521:EGFR tyrosine kinase inhibitor resistance | 5 | 1.76x10^-5^ |
| GOTERM_BP_DIRECT | GO:0010628~positive regulation of gene expression | 7 | 1.83x10^-5^ |
| KEGG_PATHWAY | hsa04012:ErbB signaling pathway | 5 | 2.35x10^-5^ |
| GOTERM_MF_DIRECT | GO:0005515~protein binding | 23 | 1.27x10^-4^ |
| KEGG_PATHWAY | hsa04910:Insulin signaling pathway | 5 | 1.52x10^-4^ |
| KEGG_PATHWAY | hsa05200:Pathways in cancer | 7 | 4.91x10^-4^ |
| GOTERM_BP_DIRECT | GO:0090090~negative regulation of canonical Wnt signaling pathway | 4 | 5.75x10^-4^ |
| KEGG_PATHWAY | hsa04151:PI3K-Akt signaling pathway | 6 | 5.99x10^-4^ |
| KEGG_PATHWAY | hsa04211:Longevity regulating pathway | 4 | 7.63x10^-4^ |
| KEGG_PATHWAY | hsa05215:Prostate cancer | 4 | 9.80x10^-4^ |
| GOTERM_MF_DIRECT | GO:0005102~receptor binding | 5 | 1.05x10^-3^ |
| KEGG_PATHWAY | hsa04066:HIF-1 signaling pathway | 4 | 1.37x10^-3^ |
| GOTERM_CC_DIRECT | GO:0070062~extracellular exosome | 9 | 1.44x10^-3^ |
| GOTERM_BP_DIRECT | GO:0045600~positive regulation of fat cell differentiation | 3 | 1.84x10^-3^ |
| KEGG_PATHWAY | hsa04152:AMPK signaling pathway | 4 | 1.86x10^-3^ |
| GOTERM_BP_DIRECT | GO:0014823~response to activity | 3 | 1.97x10^-3^ |
| GOTERM_BP_DIRECT | GO:0008286~insulin receptor signaling pathway | 3 | 2.11x10^-3^ |
| GOTERM_CC_DIRECT | GO:0031093~platelet alpha granule lumen | 3 | 2.32x10^-3^ |
| GOTERM_BP_DIRECT | GO:0042752~regulation of circadian rhythm | 3 | 2.54x10^-3^ |
| LGI3-downregulated, favorable | |  |  |
| GOTERM_BP_DIRECT | GO:0014068~positive regulation of phosphatidylinositol 3-kinase signaling | 4 | 4.12x10^-5^ |
| GOTERM_BP_DIRECT | GO:0051000~positive regulation of nitric-oxide synthase activity | 3 | 2.07x10^-4^ |
| GOTERM_CC_DIRECT | GO:0005615~extracellular space | 8 | 3.47x10^-4^ |
| GOTERM_BP_DIRECT | GO:0046427~positive regulation of JAK-STAT cascade | 3 | 4.22x10^-4^ |
| KEGG_PATHWAY | hsa04380:Osteoclast differentiation | 4 | 9.60x10^-4^ |
| KEGG_PATHWAY | hsa05418:Fluid shear stress and atherosclerosis | 4 | 1.22x10^-3^ |
| GOTERM_BP_DIRECT | GO:0010693~negative regulation of alkaline phosphatase activity | 2 | 2.48x10^-3^ |
| GOTERM_BP_DIRECT | GO:0046330~positive regulation of JNK cascade | 3 | 2.92x10^-3^ |
| KEGG_PATHWAY | hsa05417:Lipid and atherosclerosis | 4 | 4.24x10^-3^ |
| GOTERM_BP_DIRECT | GO:0051897~positive regulation of protein kinase B signaling | 3 | 5.00x10^-3^ |
| GOTERM_BP_DIRECT | GO:1903721~positive regulation of I-kappaB phosphorylation | 2 | 5.79x10^-3^ |
| GOTERM_CC_DIRECT | GO:0005886~plasma membrane | 10 | 6.77x10^-3^ |
| GOTERM_MF_DIRECT | GO:0030235~nitric-oxide synthase regulator activity | 2 | 7.13x10^-3^ |
| GOTERM_BP_DIRECT | GO:0006955~immune response | 4 | 7.19x10^-3^ |
| GOTERM_MF_DIRECT | GO:0005131~growth hormone receptor binding | 2 | 8.71x10^-3^ |
| LGI3-downegulated, unfavorable | |  |  |
| GOTERM_BP_DIRECT | GO:0010628~positive regulation of gene expression | 10 | 1.98x10^-8^ |
| GOTERM_BP_DIRECT | GO:0006468~protein phosphorylation | 9 | 2.79x10^-7^ |
| KEGG_PATHWAY | hsa04151:PI3K-Akt signaling pathway | 10 | 5.40x10^-7^ |
| KEGG_PATHWAY | hsa05200:Pathways in cancer | 11 | 1.67x10^-6^ |
| KEGG_PATHWAY | hsa05135:Yersinia infection | 7 | 2.73x10^-6^ |
| KEGG_PATHWAY | hsa01521:EGFR tyrosine kinase inhibitor resistance | 6 | 3.41x10^-6^ |
| KEGG_PATHWAY | hsa05163:Human cytomegalovirus infection | 8 | 3.46x10^-6^ |
| GOTERM_BP_DIRECT | GO:0007165~signal transduction | 11 | 4.30x10^-6^ |
| GOTERM_BP_DIRECT | GO:0008284~positive regulation of cell proliferation | 8 | 7.92x10^-6^ |
| KEGG_PATHWAY | hsa05215:Prostate cancer | 6 | 9.39x10^-6^ |
| KEGG_PATHWAY | hsa01522:Endocrine resistance | 6 | 9.88x10^-6^ |
| KEGG_PATHWAY | hsa04625:C-type lectin receptor signaling pathway | 6 | 1.32x10^-5^ |
| GOTERM_MF_DIRECT | GO:0005515~protein binding | 28 | 1.65x10^-5^ |
| KEGG_PATHWAY | hsa04062:Chemokine signaling pathway | 7 | 1.91x10^-5^ |
| GOTERM_MF_DIRECT | GO:0004713~protein tyrosine kinase activity | 5 | 1.92x10^-5^ |
| KEGG_PATHWAY | hsa05167:Kaposi sarcoma-associated herpesvirus infection | 7 | 2.02x10^-5^ |
| GOTERM_MF_DIRECT | GO:0004714~transmembrane receptor protein tyrosine kinase activity | 5 | 2.13x10^-5^ |
| GOTERM_BP_DIRECT | GO:0031295~T cell costimulation | 4 | 2.51x10^-5^ |
| GOTERM_CC_DIRECT | GO:0032991~macromolecular complex | 8 | 2.59x10^-5^ |
| KEGG_PATHWAY | hsa05213:Endometrial cancer | 5 | 2.60x10^-5^ |
